# Integrating viability and fecundity selection to illuminate the adaptive nature of genetic clines

**DOI:** 10.1002/evl3.3

**Published:** 2017-05-03

**Authors:** Susana M. Wadgymar, S. Caroline Daws, Jill T. Anderson

**Affiliations:** ^1^ Department of Genetics and Odum School of Ecology University of Georgia Athens Georgia 30602; ^2^ Department of Ecology, Evolution and Behavior University of Minnesota St. Paul Minnesota 55108

**Keywords:** Elevational gradient, flowering phenology, invisible fraction, stabilizing selection, specific leaf area, water‐use efficiency

## Abstract

Genetically based trait variation across environmental gradients can reflect adaptation to local environments. However, natural populations that appear well‐adapted often exhibit directional, not stabilizing, selection on ecologically relevant traits. Temporal variation in the direction of selection could lead to stabilizing selection across multiple episodes of selection, which might be overlooked in short‐term studies that evaluate relationships of traits and fitness under only one set of conditions. Furthermore, nonrandom mortality prior to trait expression can bias inferences about trait evolution if viability selection opposes fecundity selection. Here, we leveraged fitness and trait data to test whether phenotypic clines are genetically based and adaptive, whether temporal variation in climate imposes stabilizing selection, and whether viability selection acts on adult phenotypes. We monitored transplants of the subalpine perennial forb, *Boechera stricta* (Brassicaceae), in common gardens at two elevations over 2–3 years that differed in drought intensity. We quantified viability, and fecundity fitness components for four heritable traits: specific leaf area, integrated water‐use efficiency, height at first flower, and flowering phenology. Our results indicate that genetic clines are maintained by selection, but their expression is context dependent, as they do not emerge in all environments. Moreover, selection varied spatially and temporally. Stabilizing selection was most pronounced when we integrated data across years. Finally, viability selection prior to trait expression targeted adult phenotypes (age and size at flowering). Indeed, viability selection for delayed flowering opposed fecundity selection for accelerated flowering; this result demonstrates that neglecting to account for viability selection could lead to inaccurate conclusions that populations are maladapted. Our results suggest that reconciling clinal trait variation with selection requires data collected across multiple spatial scales, time frames, and life‐history stages.

**Impact Summary**

Natural selection has produced extraordinary diversity in adaptations to natural environments. Many species are distributed broadly across climatic gradients, such as elevation or latitude. Natural populations of these species often exhibit continuous clines in morphology and physiology in response to environmental variation. Clines provide supurb opportunities to study natural selection and adaptation. We performed a field experiment replicated across space and over time to evaluate the environmental context of clinal trait variation and natural selection in the perennial montane plant *Boechera stricta*. We transplanted *N* = 4510 individuals derived from *N* = 24 maternal families into natural communities in two experimental gardens. Over 2–3 growing seasons, we measured survival, flowering success, fruit production, and four ecologically relevant traits. By incorporating the broad range of trait values found in populations distributed across a steep environmental gradient, our experiment allowed us to test whether selection favored trait values associated with local populations. When we modeled data from all years in one analysis, we found evidence for stabilizing selection on all traits; that is, selection favored intermediate trait values, consistent with average values for local plants. In contrast, analyses for each individual year of data typically identified only weak, directional selection. Lastly, our experiment revealed that selection during juvenile stages can act on adult traits that have yet to be expressed, demonstrating the need to account for selection acting throughout a species's life cycle to characterize adaptation. Our results suggest that accurate depictions of natural selection depend on the suite of traits being examined, the duration of the experimental study, and the number of study sites under observation.

## Introdution

Adaptive evolution generates phenotypic innovation, enables species to colonize new environments, and promotes speciation (Kocher 2004; Prentis et al. 2008; Gilbert et al. 2015). Genetically based clines in functional traits can signify adaptation to continuous environmental variation along gradients, yet phenotypic divergence along gradients can also arise from plasticity and neutral processes (Vasemägi 2006; Montesinos‐Navarro et al. 2011; Kooyers et al. 2014). Testing whether genetic clines are adaptive requires evaluating selection in the natural environments in which species evolve; however, accurately estimating selection gradients can be challenging when patterns of selection vary temporally or across life history.

Environmental conditions differ across the landscape and change over time, exposing natural populations to spatial and temporal variation in selection (Grant and Grant 2002; Morrissey and Hadfield 2012; Siepielski et al. 2013; Ågren et al. 2017). Spatially variable selection can drive adaptive population divergence (Hall and Willis 2006), and temporal shifts can maintain variation within populations (Calsbeek et al. 2010). In natural populations, the presence of extensive genetic variation in ecologically relevant traits (Mousseau and Roff 1987; Paaby and Rockman 2014), in concert with pervasive directional selection, should facilitate adaptation (Kingsolver et al. 2001; Thurman and Barrett 2016) and lead to rapid evolutionary change. Paradoxically, many populations also exhibit evolutionary stasis, such that trait values do not change appreciably with time (Estes and Arnold 2007; Haller and Hendry 2014). Fluctuating selection can, theoretically, lead to evolutionary stasis if the direction of selection reverses sign frequently (Siepielski et al. 2009; Bell 2010). These changes in the sign of directional selection over time could manifest in stabilizing selection across multiple episodes of selection as populations repeatedly traverse the summit of a fitness peak (Phillips and Arnold 1989; McGlothlin 2010). However, natural populations that appear highly adapted to contemporary conditions often show evidence for directional, not stabilizing, selection in studies that evaluate only one episode of selection (Kingsolver and Diamond 2011; Kingsolver et al. 2012). Evolutionary stasis will arise if the position of the peak within phenotypic space is stable (Arnold et al. 2008). Therefore, temporal variation in selection could explain the stability of genetic clines despite frequent obsevations of short‐term directional selection.

Evidence for temporally varying selection in wild populations is limited (Morrissey and Hadfield 2012). Subtle fluctuations in linear selection gradients may be the only indicators of nonlinear selection, as stabilizing selection can be difficult to detect directly when sample sizes are low or populations inhabit a broad or stable fitness peak (Haller and Hendry 2014). By incorporating a wider range of phenotypes than typically found in a single population, multiyear common garden experiments provide powerful tests of the role of temporal variation in generating stabilizing selection over time, particularly in perennial species for which trait evolution is influenced by multiple episodes of selection. Furthermore, replicate gardens across environmental gradients can reveal whether selection maintains clinal trait variation.

Detecting selection is particularly challenging when a nonrandom portion of individuals die prior to trait expression (Hadfield 2008). Nevertheless, studies that neglect viability selection could generate biased estimates of selection and inaccurate predictions about trait evolution (Hadfield 2008; Mojica and Kelly 2010). For example, despite fecundity selection favoring large flowers, low survival of large‐flowered *Mimulus guttatus* genotypes drives selection for smaller flowers via lifetime fitness (Mojica and Kelly 2010). Mortality events generally prevent experiments from retaining sufficient statistical power to estimate viability selection. Multivariate genotypic selection analyses (Rausher 1992) are well‐suited for evaluating selection because they account for missing data generated from mortality (i.e. the “invisible fraction”) by modeling family‐level trait and fitness data (Hadfield 2008).

We conducted a multiyear field study to disentangle the conributions of adaptation, plasticity, and neutral evolutionary processes to clinal trait variation and to characterize the nature of selection on functional traits in the context of spatiotemporal environmental variation. Elevation gradients present superb opportunities for investigating adaptation and spatial variation in selection. Climatic conditions change appreciably over short spatial scales in mountainous systems (Körner 2007), which can result in consistent patterns of adaptation to local climate (Byars et al. 2007; Kim and Donohue 2013). Indeed, populations of the subalpine perennial forb *Boechera stricta* (Brassicaceae) experience spatially restricted gene‐flow and have adapted locally to variation in selective regimes that occur over relatively short spatial scales (Anderson et al. 2015).

We hypothesize that (1) genetically based clines in flowering phenology, morphology, and ecophysiology are consistent with clinal trait variation in natural populations (Anderson and Gezon 2015) and are maintained by selection. By assessing spatial and temporal patterns of natural selection, we tested whether (2) natural selection fluctuates around local phenotypic optima. Finally, we estimated selection at two life‐history stages to evaluate whether (3) viability selection operating prior to trait expression influences the evolutionary trajectories of adult phenotypes. Despite numerous estimates of selection in nature (Kingsolver et al. 2012), studies rarely examine spatial and temporal dynamics simultaneously (Siepielski et al. 2013), especially across multiple life‐history stages.

## Methods

### SYSTEM

We conducted fieldwork in subalpine meadows around the Rocky Mountain Biological Laboratory (RMBL, Gunnison County, Colorado). *Boechera stricta* is a short‐lived, self‐fertilizing perennial forb native to the Rocky Mountains, where it occurs across a broad elevational gradient (700–3900 m) (Al‐Shehbaz and Windham 2010). We estimate the generation time of this species to be 2–3 years (Anderson et al. 2012) and individuals experience multiple episodes of selection across their lifetimes. In Gunnison county and other regions in Colorado, robust populations of *B. stricta* occur from ∼2700 m to ∼3600 m in elevation, with sparse populations in elevations as low as 2500 m, but we have not located populations at lower elevations (Anderson, pers. obs.). High elevation sites have later spring snowmelt, cooler temperatures, greater water availability, and shorter growing seasons than low elevation locales (Dunne et al. 2003; Anderson and Gezon 2015).

Our study captured striking differences in climate from 2012–2014 (Harte et al. 2015). These years followed a sequence of decreasing abiotic stress, with snowfall levels of 640, 788, and 1177 cm from 2012 to 2014, and snowmelt occurring earlier than the historical average by 44 days, 23 days, and 13 days in those same years. Snowfall averaged 1088.3 ± 273.0 cm annually from 1974–2015 (mean ±SD; b. barr, Gothic Long‐Term Weather Data; http://www.gothicwx.org/long-term-snow.html) (Harte et al. 2015).

### COMMON GARDENS

To test unresolved hypotheses about natural selection and genetic clines, we transplanted maternal families collected across a broad elevational gradient into two common gardens: a hot and dry low‐elevation site (2891 m, 38°57.086”N, 106°59.4645”W) and a cool and wet high‐elevation site (3133 m, 39°02.346”N, 107˚03.818”W). As expected based on global and regional patterns (Körner 2007), weather stations near our field sites show that mean annual temperature declines by 0.0023–0.0046°C and total annual precipitation increases by 0.042 cm for every meter gain in elevation (Anderson and Gezon 2015). Snow melted 11 days earlier in the low versus high elevation garden in 2013 and 2014, and Decagon 5TM sensors installed in 2014 showed that soil temperature was 4.52°C ± 0.04 greater in the lower versus higher garden from July 2–August 28, 2014 (Anderson and Gezon 2015).

We examined selection on four traits associated with climatic adaptation: specific leaf area (SLA), stable Carbon isotopes (δ^13^C), height at first flower, and flowering phenology (e.g., Campbell et al. 2010; Ward et al. 2012; Pratt and Mooney 2013; Read et al. 2014; Ågren et al. 2017). Natural populations of *B. stricta* exhibit significant phenotypic clines in these ecologically relevant traits across elevational gradients (Anderson and Gezon 2015). Foliar stable carbon isotopes (δ^13^C) provide robust data on water‐use efficiency (WUE) integrated over the lifespan of the leaf (Farquhar et al. 1989). In regions such as the Colorado Rockies where aridity and growing season length decrease with elevation, we expect selection to favor drought tolerance at low elevations and rapid development at high elevations, in which case WUE (δ^13^C) and flowering time would decline with source elevation. However, similar clines in δ^13^C could emerge from declining partial pressure of atmospheric CO_2_ with elevation (Körner 2007).

The power to test adaptive population divergence is greater in studies that include few families from a large number of populations, rather than studies that include more families from fewer populations (Goudet and Buchi 2006; Blanquart et al. 2013). Therefore, we selected one maternal family from each of 24 local populations, which ranged in elevation from 2869–3682 m (Table S1), enabling tests of selection using accessions that experienced substantially different environmental conditions during their evolutionary histories (Anderson and Gezon 2015; Anderson et al. 2015). For the 24 source populations, we extracted climatic data from WorldClim using a bilinear interpolation with the highest spatial resolution available (30 arc‐second). In accordance with data from sensors installed within our field sites, temperature, and aridity decline with elevation (*R* ^2^ = 0.985 and 0.96, respectively, *p* < 0.0001), while precipitation increases with elevation (*R* ^2^ = 0.96, *p* < 0.0001). Therefore, source elevation can serve as a reliable proxy for climate of origin in this region.

Since *B. stricta* self‐pollinates and has limited within population genetic variation (Song et al. 2006), our sampling design maximized genetic diversity and is effective for quantifying genetic clines and selection. This experiment has revealed local adaptation to elevation and genetic isolation by distance (Anderson et al. 2015) as well as plasticity in foliar morphology and genetically based clines in flowering phenology in the 2013 growing season (Anderson and Gezon 2015). Here, we integrate trait and fitness data from a larger database covering 2012–2014 to assess temporal variation in genetic clines and selection on phenology, morphology, and ecophysiology.

To reduce maternal effects and generate families via self‐fertilization, we grew field‐collected seeds for one generation in the greenhouse and allowed them to self‐pollinate. We then germinated seeds from these full sibling maternal lines and reared seedlings in the greenhouse for three months prior to outplanting. In September 2011, we transplanted *N* = 2293 juvenile plants into the low elevation garden (hereafter: 2011 cohort; ∼95.5 full siblings per family). In September 2012, we transplanted a second cohort into both gardens (hereafter: 2012 cohort): *N* = 1096 individuals into the lower garden and *N* = 1121 individuals into the higher garden (∼46 full siblings per family per garden). We used seeds of the same generation and the same 24 families in both cohorts and gardens, and planted two full siblings per family into blocks of 48 individuals. Extensive replication of siblings within each family allowed us to generate precise and accurate estimates of heritabilities, family‐level trait means, and ultimately selection gradients. We disturbed the natural community minimally during transplanting and did not manipulate the biotic or abiotic environment. This experiment captured the full lifespan of most experimental individuals, as only 21.9% (2011 cohort) and 49% (2012 cohort) of individuals remained alive in the final census (September 2014).

### FITNESS COMPONENTS AND PHENOTYPES

In May–August 2012–2014, we visited each garden 3–4 times/week and recorded survival, plant size, numbers of flowers and fruits, and the length of the longest fruit. We collected all mature fruits. For plants that flowered between censuses, we estimated the day of first flowering from data on the number of fruits and fruit elongation rates (Table S2). Analysis of the raw flowering time data (not shown) generated similar results. We calculated flowering time as the number of elapsed days between snowmelt and first flowering. Fitness components included flowering success (viability) and fecundity. At the end of each season, we calculated the sum of the lengths of all mature fruits for each plant, excluding any aborted fruits lacking seeds. We used total mature fruit length as our metric of fecundity, as it correlated with seed number (Pearson correlation coefficient: *r* = 0.71, *p* < 0.0001, *N* = 72) and weight (*r* = 0.74, *p* < 0.0001, *N* = 72).

We quantified integrated water‐use efficiency via natural abundance stable Carbon isotopes (δ^13^C) using samples from ∼3 siblings per family collected each year from both gardens (Table S3). We pulverized leaf tissue using a GenoGrinder (SPEX SamplePrep), weighed 3.000–3.200 mg of ground foliar tissue on an ultramicrobalance (UMX2, Mettler‐Toledo) and loaded tissue into tin capsules. The Cornell University Stable Isotope Laboratory combusted samples on an isotope ratio mass spectrometer connected to an elemental analyzer. We report δ^13^C relative to Vienna Pee Dee Belemnite. Traits in this study were moderately correlated (|*r*| < 0.3, Table S3).

### ANALYSES

#### Genetic variation

In *B. stricta* and other selfing species, responses to selection depend on total genetic variance rather than additive genetic variance (Roughgarden 1979). We used restricted maximum likelihood to estimate broad‐sense heritability (H^2^) for each garden and cohort as genetic variance (V_G_) divided by phenotypic variance (V_P_ = family variance + block variance + error variance) in models that included random effects for family and block and repeated effects for plant identity across seasons (Proc Mixed, SAS ver. 9.4). To estimate the heritability of flowering success, we used a binary distribution with a logit link (Proc Glimmix).

#### Genetically based clines and plasticity

To assess genetic clines and plasticity, we modeled all traits simultaneously in a repeated measures multivariate regression with a Kenward‐Roger degree of freedom approximation. We analyzed the two cohorts separately because we have three years of data from one garden (2011 cohort) and two years of data from both gardens (2012 cohort). We first calculated family level averages (LSMEANS) by modeling each trait in each season as a function of fixed effects for family (2011 cohort) or family × garden (2012 cohort) with a random effect for block (Proc Mixed). We then analyzed multivariate family‐level LSMEANS as a function of source elevation, season, and source elevation × season (full results in Tables S4 and S5). For the 2012 cohort, we also included the main effect of garden, as well as 2‐ and 3‐way interactions among garden, source elevation, and season. Analyses of individual‐level data generated quantitatively similar results (Table S6). Multivariate repeated measures models use direct (Kronecker) product structures to specify the covariance structure of the R matrix [type = UN@AR(1)], which fits multiple response variables (unstructured covariance matrix, UN) measured on the same plants across years [autoregressive covariance matrix, AR(1)] (Galecki 1994). We standardized trait values to a mean of 0 and standard deviation of 1 but present unstandardized data in figures.

We directly estimated genetic clines by incorporating source elevation of maternal families into our analyses. Interactions between garden or season and source elevation indicate that genetic clines vary across space or through time. We used preplanned estimate statements to calculate the slope and test the significance of genetic clines for each trait, controlling for multiple tests with the Benjamini–Hochberg procedure ( 1995); we report corrected *P*‐values in the results. Significant main effects of season and garden reveal temporal and spatial plasticity, respectively. Table S6 contains complementary analyses correcting for neutral population genetic structure using 13 microsatellite loci genotyped previously (Anderson et al. 2015).

#### Genotypic selection analyses

To test whether clines are adaptive, we used genotypic selection analyses (Rausher 1992). As traits and fitness were highly heritable (Table S3), we can link family‐level trait data (averaged over individuals that expressed traits) with family‐level fitness (averaged over all individuals, including those that died or failed to reproduce). These analyses test if selection operates prior to trait expression by incorporating the invisible fraction into fitness components (Hadfield 2008). We analyzed selection via three fitness components: probability of flowering among plants alive at the beginning of each season (viability), the length of fruit (a proxy for seed set) among plants that successfully flowered (fecundity), and fruit length among all individuals (cumulative). Genotypic selection analysis and artificial selection experiments are currently the only mechanisms to evaluate viability selection when individuals die before trait expression (Hadfield 2008). Our estimated selection gradients are likely conservative because the most maladapted individuals probably die earlier, such that their (maladapted) trait values would not contribute to family mean phenotypes.

To investigate whether selection fluctuates around local phenotypic optima, we conducted genotypic selection analyses using Aster models (Geyer et al. 2007). Aster assesses lifetime selection by modeling the dependence of fitness components on those expressed earlier in life history (Shaw et al. 2008). This approach can integrate episodes of selection throughout the life cycle, allowing us to compare the contributions of selection acting through viability and fecundity to cumulative patterns of selection (Table S7).

To the best of our knowledge, Aster analyses have not yet been used to conduct genotypic selection analyses. Although able to integrate multiple episodes of selection, Aster models discard data for families with incomplete trait data for any year, resulting in five dropped families from the 2011 cohort and two from the 2012 cohort. Data from maternal families that fail to reproduce in a given year are inherently valuable for quantifying viability selection. As such, we have replicated all analyses in a repeated measures framework in SAS, which only removes families from the specific years with missing trait data. SAS models resulted in more conservative estimates of selection as compared to Aster in some cases. We treat the few discrepancies between results generated from both platforms as tentative (see Supplemental Materials for details on Aster and SAS methodology).

We conducted cumulative, viability, and fecundity selection analyses separately for each cohort, evaluating direct selection by analyzing fitness as a function of all four traits simultaneously. We included source elevation as a covariate in in our models to account for unmeasured traits that may also vary across elevational gradients, and we evaluated quadratic effects of source elevation through loglikelihood tests (for a similar approach, see Colautti and Barrett 2013). To test for spatial variation in selection, we included fixed effects for garden and trait × garden interactions in analyses of the 2012 cohort. Significant interactions indicate that selection varies spatially, in which case we extracted selection gradients from overall Aster models, but estimated the significance of selection gradients in separate models for each garden. We then analyzed each growing season separately in SAS models (Proc Glimmix and Proc Mixed, Table S11). We standardized all traits to a mean of 0 and standard deviation of 1 and we relativized fecundity and cumulative fitness to a mean of 1. We converted logistic regression coefficients to selection gradients following Janzen and Stern (1998). We estimated standardized linear selection gradients and statistical significance in models that contained linear effects only; we extracted quadratic selection gradients and significance from second‐order polynomial models including linear and quadratic effects of traits (and other relevant fixed effects) (Lande and Arnold 1983). We doubled all quadratic regression coefficients and standard errors to estimate quadratic selection gradients (Stinchcombe et al. 2008).

## Results

Phenotypes and fitness components were highly heritable in this study, with H^2^ ranging from 0.083–0.43 for the 18 estimates of heritability (average: 0.25; all *P* < 0.0001 after correction for multiple testing; Table S3). We summarize predictions and results in Table 1.

**Table 1:** Predictions and results for genetic clines, plasticity, and selection

Trait	Predicted: genetic cline	Predicted: plasticity	Results: genetic clines	Results: plasticity	Results: selection and clines	Results: viability vs fecundity
Water‐use efficiency (δ^13^C)	(–)	Greater trait values in dry environments (low vs high elevation)	Concordant and context dependent	Concordant	Concordant	No
Specific leaf area (cm^2^/g)	(+)*	Lower trait values in dry environments (low vs high elevation)	Concordant and context dependent	Concordant	Concordant	No
Flowering time (relative to snowmelt date)	(–)	Earlier flowering under short seasons (high vs low elevation)	Concordant and context dependent	Concordant	Concordant	Yes
Height at flowering	(–)	Shorter height at flowering in high elevations	Concordant and context dependent	Concordant	Concordant	No

We base predictions are on phenotypic variation across natural *Boechera stricta* populations (Anderson and Gezon 2015) and on climatic adaptation from other systems (e.g., Campbell et al. 2010; Leonardi et al. 2012; Ward et al. 2012; Pratt and Mooney 2013; Read et al. 2014). “Predicted: Genetic cline” indicates the predicted relationship between trait values and source elevation in common gardens, with (–) predicting a negative slope and (+) predicting a positive slope. “Predicted: Plasticity” provides expectations for trait variation with environment. In the two corresponding results columns, we indicate whether genetic clines and phenotypic plasticity were concordant or discordant with predictions. “Results: Selection and clines” conveys whether selection is generally concordant with genetic clines, with fitness optima integrated across time and life history corresponding to local mean trait values. Lastly, “Results: Viability vs fecundity” illustrates whether viability and fecundity selection favor contrasting trait values.

^*^Note: This prediction is based on natural *B. stricta* populations (Anderson and Gezon 2015). Other systems show the reverse pattern (see discussion and Read et al. 2014).

### GENETICALLY BASED CLINES ARE CONSISTENT WITH CLINAL TRAIT VARIATION IN NATURAL POPULATIONS

**Figure 1:**
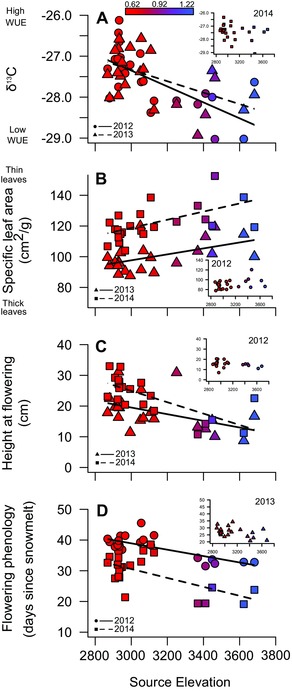
Genetically based clines in four functional traits measured over three growing seasons for the 2011 cohort planted into the low elevation garden: (A) Carbon isotope discrimination (integrated water‐use efficiency, WUE), (B) specific leaf area, (C) height at flowering, and (D) flowering phenology. Data points represent family‐level averages for these heritable traits. Lines depict the shape of the association between source elevation and trait values (only shown for significant relationships after correction for multiple tests). Inset panels reflect seasons in which we found no significant genetic clines. Figure S1 presents genetically based clines for the 2012 cohort. The color coding represents calculated Aridity Index based on climatic data extracted from WorldClim, ranging from arid low elevation populations (red) to less arid high elevation populations (blue).

In both gardens, we detected significant genetically based clines in traits, which were consistent with the direction and magnitude of trait variation across elevation in natural populations (Anderson and Gezon 2015) and were generally robust to correction for neutral population differentiation (Tables S4–S6, Figs. 1 and S1). Furthermore, genetic clines were concordant with spatial plasticity in these traits (Table S4, Fig. S2). Nevertheless, genetic clines did not emerge in all years. In accordance with our predictions (Table 1), integrated WUE (as measured via stable Carbon isotopes, δ^13^C) declined significantly with source elevation in 2012, the year of extreme early snowmelt (*t*_51.6_ = –3.65, *P* = 0.0019; Fig. 1A; Table S5). This genetic cline persisted when drought was less severe in 2013 (*t*_51.6_ = –2.62, *P* = 0.017), but disappeared in the relatively benign year of 2014 (*t*_51.6_ = –1.23, *P* = 0.24; Fig. 1 inset). Context dependency became even more apparent after correcting for neutral population structure, as the genetic cline was only significant for the driest year of 2012 (Table S6). The 2012 cohort showed no evidence for genetic clines in δ^13^C (Table S5B, Fig. S2).

Genetic clines in specific leaf area (SLA) were concordant with trait variation in natural *B. stricta* populations (Anderson et al. 2015): SLA increased significantly with source elevation in two years for the 2011 cohort (2013: *t*_61.2_ = 2.74, *P* = 0.014; 2014: *t*_61.2_ = 3.69, *P* = 0.0019, Table S5; Fig. 1B), and we saw a similar positive relationship in the lower garden of the 2012 cohort in 2014 (*t*_82.6_ = 2.63, *P* = 0.026, Fig. S1 and Table S5). In the higher garden, there was a marginal trend in the same direction in 2013 (*t*_84.5_ = 2.25, *P* = 0.053). Clines in SLA were nonsignificant after correcting for neutral population structure (Table S6), yet we found evidence that selection favors local trait values in both cohorts (see below).

Growing seasons are limited in duration in high elevation sites; therefore, we predicted that plants in high elevation locales would flower rapidly at small stature to complete their reproductive cycles prior to the onset of inclement conditions. Genetic clines were consistent with expectations and generally stable across space and time. For the 2011 cohort, we saw the expected decline in flowering time with source elevation in two years (2012: *t*_52.6_ = –3.22, *P* = 0.0052; 2013: *t*_52.5_ = –1.7, *P* = 0.11; 2014: *t*_53.9_ = –4.34, *P* = 0.0004, Fig. 1C, Table S5) and the expected reduction in height at flowering with source elevation in two of the three years (2012: *t*_57.1_ = –0.68, *P* = 0.50; 2013: *t*_57_ = –2.86, *P* = 0.012; 2014: *t*_58.1_ = –4.79, *P* = 0.0001, Fig. 1D). In three of four cases, the clines in size at reproduction and flowering time are also apparent in the 2012 cohort (Table S5, Fig. S1). Reproductive phenology clines generally remain significant after correction for neutral population structure (Table S6).

#### Natural selection

(1) Selection fluctuates around local phenotypic optima and (2) viability selection influences adult trait evolution

**Figure 2:**
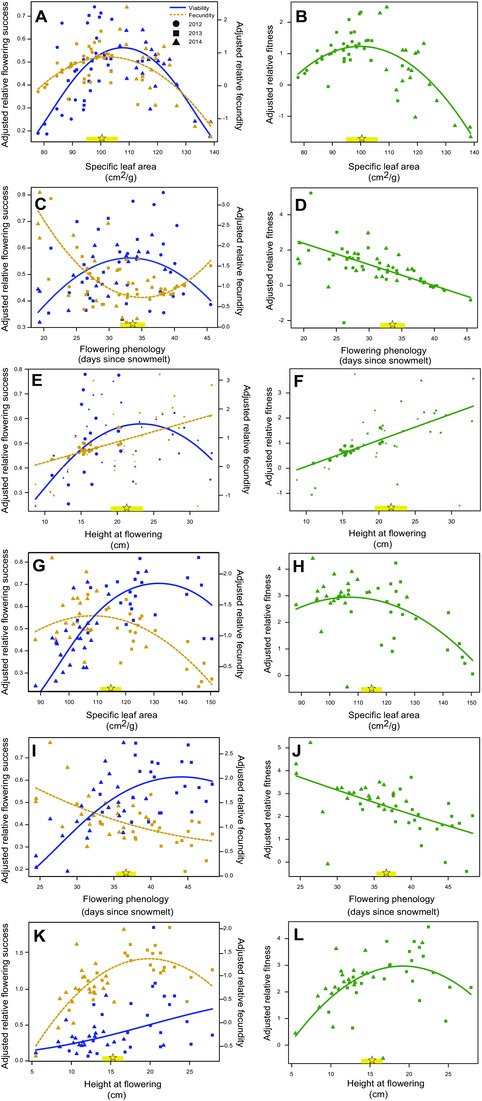
Patterns of viability and fecundity selection (left column) and the resulting cumulative selection (right column) on (A, B) SLA in the 2011 cohort, (C, D) flowering phenology in the 2011 cohort, (E, F) height at flowering in the 2011 cohort, (G, H) SLA in the 2012 cohort from the high‐elevation garden, (I, J) flowering phenology in the 2012 cohort from the high‐elevation garden (2012 cohort), and (K, L) height at flowering in the 2012 cohort from high‐elevation garden. These analyses include data from all years of the study. Y‐axes display adjusted fitness values, which were statistically corrected for other variables included in the models by adding residuals from full models to predicted fitness values estimated from regression coefficients for the trait of interest. We depict fitness curves using linear and (undoubled) quadratic regression coefficients from multivariate models, but we doubled quadratic regression coefficients to calculate nonlinear selection gradients in Tables S8 and S10 (Stinchcombe et al. 2008). Mean trait values for local genotypes are displayed with a yellow star and are bracketed in yellow by 2 × SE. Local values were extracted from models of genetically based clines (Figs. 1 and S1) for families with a source elevation of 2891 m (the elevation of the lower garden) or 3133 m (the elevation of the higher garden).

We evaluated direct selection via three fitness components: the probability of flowering (viability selection), fruit length (a proxy for seedset) among plants that flowered (fecundity selection), and fruit length among all plants (cumulative selection). Temporal fluctuations in environmental conditions alter not only the distribution of phenotypes in our study (Fig. S2 and Table S4), but also the magnitude and direction of selection. Indeed, multivariate genotypic selection analyses uncovered significant linear and quadratic direct selection. Local families typically expressed trait values close to trait optima, suggesting that genetic clines are adaptive (Fig. 2). Furthermore, nonlinear selection was primarily apparent in models that integrated data across years rather than models investigating selection separately for each growing season (Tables S8–S12).

#### 2011 cohort

Specific leaf area was under stabilizing selection across life history, with similar intermediate trait optima favored by viability and fecundity selection (viability: χ^2^(1) = 32.7, *P* < 0.0001; fecundity: χ^2^(1) = 55.2, *P* < 0.0001; Fig. 2A). The synergy between viability and fecundity selection resulted in cumulative selection favoring SLA values similar to the phenotypes of local families (χ^2^(1) = 7.51, *P* = 0.006; Fig. 2B).

Patterns of selection on the other vegetative trait (δ^13^C) were not as consistent. Aster models detected stabilizing viability selection favoring intermediate δ^13^C values (χ^2^(1) = 7.21 *P* = 0.007, Fig. S3A), but SAS failed to find evidence for significant viability selection on this trait (Table S8). Additionally, Aster models uncovered weak nonlinear significant selection on δ^13^C via fecundity (χ^2^(1) = 9.4, *P* = 0.002, Fig. S3A), which again was not evident in SAS analysis (Table S8). As SAS was unable to detect selection on δ^13^C, we treat these Aster results as tentative.

**Figure 3:**
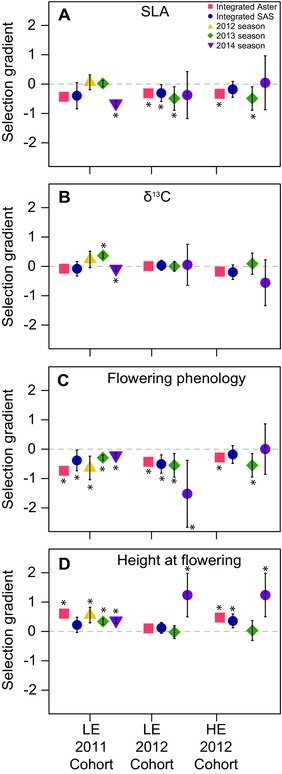
Direct linear selection gradients from cumulative selection analyses in the 2011 and 2012 cohorts in the low elevation (LE) and high elevation (HE) gardens for (A) SLA, (B) carbon isotope discrimination (integrated water‐use efficiency, WUE), (C) flowering phenology, and (D) height at flowering. We show the direction and magnitude of selection from analyses integrated across years (Aster and SAS models) as well as from separate analyses for each season of study (SAS models). Estimating standard errors from Aster models is not straightforward. Thus, stars for integrated Aster models indicate significant selection as revealed by log likelihood ratio tests (Table S7–S12).

Mulitivariate models revealed stabilizing viability selection on both adult traits, with optimal trait values corresponding with phenotypes of local families (flowering phenology: χ^2^(1) = 8.07, *P* = 0.005, Fig. 2C; height at flowering: χ^2^(1) = 8.27, *P* = 0.004; Fig. 2E, Table S8). In contrast, nonlinear fecundity selection showed curvilinearity in directional selection for earlier flowering (χ^2^(1) = 17.4, *P* < 0.0001; Fig. 2C). Directional fecundity selection favored taller plants at flowering (χ^2^(1) = 32.6, *P* < 0.0001; Fig. 2E). Finally, consistent with other studies, directional cumulative selection favored earlier flowering (χ^2^(1) = 9.08, *P* = 0.003, Figs. 2D, 3C) and greater height at flowering (χ^2^(1) = 8.1, *P* = 0.005, Fig. 2F, 3D).

#### 2012 cohort

As with the 2011 cohort, stabilizing selection emerged principally in models that integrated across episodes of selection. Viability and fecundity selection on specific leaf area (SLA) varied spatially (Table S10). In the high elevation garden, stabilizing selection operated on SLA consistently across life history, with viability, fecundity, and cumulative selection favoring slightly different intermediate trait optima (viability: χ^2^(2) = 8.78, *P* = 0.012; fecundity: χ^2^(1) = 189.8, *P* < 0.0001; cumulative: χ^2^(1) = 32.4, *P* < 0.0001; Fig. 2G, H, Table S9). In contrast, in the lower garden, selection favored reduced SLA across all fitness components, although weak nonlinear selection via viability and fecundity indicated slight curvilinearity in patterns of directional selection (viability: χ^2^(1) = 8.78, *P* = 0.012; fecundity: χ^2^(1) = 116.7, p < 0.0001; linearcumulative: χ^2^(2) = 26.4, *P* < 0.0001; Table S9, Fig. S3 C, D).

Viability models detected spatially variable nonlinear selection on water‐use efficiency (WUE) as measured through δ^13^C (χ^2^(1) = 22.9, *P* < 0.0001, Table S10), with positive quadratic selection in the lower elevation garden favoring greater WUE values than expressed in local families (χ^2^(1) = 7.3, *P* = 0.012, Fig. S3E) and stabilizing selection for WUE values similar to those of local families in the higher elevation garden (χ^2^(1) = 3.94, *P* = 0.047, Fig. S3I). This pattern was counterbalanced by fecundity selection for reduced WUE in the high elevation garden (χ^2^(1) = 76.4, *P* < 0.0001, Fig. S3I), but there was no evidence for fecundity selection in the lower garden. Models detected cumulative stabilizing selection on WUE favoring local trait values in the higher elevation garden (χ^2^(1) = 16.2, *P* < 0.0001, Fig. S3J), but no evidence for cumulative selection on WUE in the lower garden.

As with the 2011 cohort, multivariate models revealed viability selection acting on adult traits. Viability selection favored delayed flowering time in the higher elevation garden (χ^2^(1) = 7.91, *P* = 0.005) while other fitness components displayed the more customary pattern of selection for earlier flowering with moderate curvilinearity (fecundity: χ^2^(1) = 36.5, *P* = 0.0001; cumulative: χ^2^(1) = 7.31, *P* = 0.007; Fig. 2I, J). In the lower elevation garden, selection was congruent across fitness components, favoring earlier flowering phenology at all life stages from viability (slight curvilinearity; χ^2^(1) = 7.58, *P* = 0.006) to fecundity (slight curvilinearity χ^2^(1) = 45.2, *P* < 0.0001) and cumulative fitness (linear relationship: χ^2^(2) = 15.1, *P* < 0.001, Fig. 3C, Fig. S3G, H). However, the viability results should be treated as tenative as SAS reported viability selection for delayed flowering even in the lower garden (β = 0.44 ± 0.16, *t*_58_ = –2.7, *P* = 0.009; Table S9).

Height at flowering was also subject to spatially variable linear selection (χ^2^(1) = 52.6, *P* < 0.0001), as viability selection favored taller plants at flowering in the higher elevation garden (Fig. 2K), but did not operate on this trait in the lower garden (Fig. S3K, Table S9). Stabilizing fecundity selection for greater size at flowering differed in magnitude across the two sites (χ^2^(1) = 63.8, *P* < 0.0001), being stronger in the higher elevation garden (Fig. 2K) than the lower garden (Fig. S3K). Cumulative selection also varied spatially (χ^2^(1) = 32.1, *P* < 0.0001), with strong stabilizing selection in the high garden (Fig. 2L), but only weak linear selection in the lower garden (Fig. S3L).

For both cohorts, separate analyses of each year revealed weaker patterns of natural selection with less evidence for stabilizing selection than models of datasets combined across years (Fig. 2, Fig. S4, Table S12). Furthermore, in the few cases where we detected stabilizing selection in separate analyses of each growing season, local trait values lie farther away from the optima than in integrated analyses, as seen for cumulative selection on SLA (Fig. 2, Fig. S4).

## Discussion

Natural selection drives adaptive evolution; however, we have yet to reconcile the stability and extent of clinal trait variation with patterns of selection. In our study, *Boechera stricta* exhibited significant genetically based clines in ecophysiological, morphological, and phenological traits that are likely maintained by selection. In all cases, the direction of genetic clines and spatial plasticity was concordant with a priori expectations based on trait variation in natural populations (Anderson and Gezon 2015), and selection often favored trait values expressed by local families. Genetically based clines likely evolved in response to long‐standing spatial variation in selection across steep elevational gradients. Here, we discuss three major findings that advance our ability to characterize adaptation in heterogeneous environments: Genetically based clines are context dependent, temporally fluctuating selection can favor intermediate phenotypic optima, and viability selection prior to trait expression can influence the evolution of adult phenotypes.

### CONTEXT DEPENDENCY OF GENETICALLY BASED CLINES

Changing environmental conditions can impose novel selection, altering the shape of genetic clines in ecologically relevant traits (e.g., Brakefield and de Jong 2011). In our study, genetic clines occurred in the same direction relative to source elevation in both gardens and cohorts, but some clines only emerged in years with limited snowpack, early snowmelt, and dry growing seasons (integrated water‐use efficiency, δ^13^C). For other traits (specific leaf area and height at flowering), genetic clines only became apparent in more benign years. Laboratory experiments may not manipulate the agents of selection that shape or maintain clinal trait variation, and the magnitude of selection can differ between lab and field environments (Kellermann et al. 2015). Thus, clinal variation may not manifest under benign controlled conditions or short‐term field studies. This context‐dependency in genetic clines and trait expression indicates that multiyear and multisite investigations in natural settings are critical for characterizing natural selection and adaptation.

#### Adaptive context of genetically based clines

Life‐history information and data from common gardens can be useful for discerning the adaptive context of genetic clines. Congruent with expectations from studies of other speices along elevational and latitudinal gradients (Monty and Mahy 2008; Kawakami et al. 2011; Woods et al. 2012, but see Stinchcombe et al. 2004), we found consistent genetic clines in phenological traits, indicating that families from low elevation populations consistently flower later and at larger sizes than high elevation families. Tradeoffs between age and size at flowering likely constrain the joint evolution of these traits (e.g., Anderson et al. 2011). In concert, these genetic clines suggest evolutionary responses to short growing seasons in populations inhabiting cooler poleward latitudes and upslope elevations.

The warmer and drier conditions at low elevation sites can favor traits that improve drought‐resistance. In *Boechera stricta*, steep (in 2012) to nonexistant (in 2014) genetic clines in δ^13^C likely reflect differential water‐use efficiency (WUE) across the elevational range in this region, as is true in other systems in which aridity declines with elevation (e.g., Lajtha and Getz 1993; Van de Water et al. 2002; Reed and Loik 2016). Had δ^13^C clines been driven primarily by partial pressure of CO_2_, we would have expected the clines to be stable across space and time. Our data suggest that low‐elevation genotypes of *B. stricta* exhibit high WUE, as is the case in the model plant *A. thaliana* (Wolfe and Tonsor 2014). Furthermore, stabilizing selection favored relatively low water‐use efficiency in the high elevation garden. Specific leaf area (SLA) increased with source elevation in our common gardens (this study) and across elevation in natural populations (Anderson and Gezon 2015). Although genetic clines in SLA were not robust to correction for neutral population genetic structure, selection favored local trait values in both gardens. This discrepancy indicates that the adaptive nature of clinal trait variation may only become evident after analysis of selection at relevant life‐history stages. Our data also suggest that high elevation *B. stricta* families invest in rapid resource acquisition because of short growing seasons, while low elevation families have evolved drought tolerance (this study) and resistance to insect herbivory (Anderson et al. 2015) because of extended dry periods and abundant herbivores. These results highlight the potential for complex suites of conditions to contribute to trait clines and selection regimes across elevational gradients.

### FLUCTUATING SELECTION FAVORS INTERMEDIATE TRAIT VALUES

In fluctuating environments, the evolutionary trajectory of a population will depend on the cumulative effects of variable selection regimes (Bell 2010). Our study was replicated for just three years, and yet we still detected temporally fluctuating selection that culiminated in strong patterns of stabilizing selection on both juvenile and adult traits. In contrast, analyses considering each year of data separately indicated that patterns of selection were generally weak and variable. Studies that neglect or underestimate fluctuating selection can falsely conclude that traits are not targeted by selection or erroneously inflate expected evolutionary responses to selection. Our data demonstrate that examining natural selection over the course of a single growing season may uncover patterns and generate predictions that are at odds with those derived from multiyear studies. By conducting our study over multiple growing seasons, we were able to quantify how temporal environmental variation shifts phenotypic distributions and components of fitness, and how these shifts alter the selective landscape.

The literature provides mixed evidence for temporal variability in directional selection. Selection on beak size and shape oscillated over three decades of observations in Darwin's finches (Grant and Grant 2002), but comparatively long‐term datasets are exceedingly rare. In a review of 5519 estimates of selection from 89 temporally replicated studies, only 25% of linear selection gradients fluctuated in sign over time (Siepielski et al. 2009). Furthermore, temporal fluctuations were primarily found for traits that experienced relatively weak selection (Kingsolver et al. 2012), with sampling error inflating heterogeneity in most estimates of selection (Morrissey and Hadfield 2012). While current empirical evidence suggests that temporal variation in selection may not be ubiquitous, it undoubtedly maintains phenotypic and genetic variation in some systems. Considering data from only one season in a study of selection can obscure the insights gained from a more integrated approach (McGlothlin 2010).

### VIABILITY SELECTION AND THE INVISIBLE FRACTION

Mortality events can present considerable challenges when characterizing natural selection (Hadfield 2008; Nakagawa and Freckleton 2008). This mortality‐generated missing data, known as the "invisible fraction," can be nonrandom with respect to ontogeny, habitat of origin, experimental treatments, and size classes (Bennington and McGraw 1995; Sinervo and McAdam 2008; Engen et al. 2012). However, most studies of natural selection do not account for viability selection, either because the family structure of focal individuals is unknown or because early survival and trait data are unavailable (but see Mojica and Kelly 2010; Tarwater and Beissinger 2013).

By monitoring survival to reproduction, we determined that viability selection targeted flowering onset and size at flowering. Furthermore, viability selection can both enforce and oppose fecundity selection. For instance, for the 2012 cohort in the high elevation garden, viability selection favored delayed flowering, and larger plant size while fecundity selection favored earlier flowering and an intermediate plant size. The onset of flowering often coincides with a shift from resource investment in vegetative growth toward reproduction. The potential fitness tradeoff between flowering early for reproductive assurance and flowering at a larger size for increased resource acquisition has been studied in relation to fruit or seed production (Bolmgren and Cowan 2008). Our results suggest that viability selection can exacerbate or alleviate this tradeoff, depending on the environmental context.

The evolution of reproductive phenology appears to represent a compromise between the opposing forces of viability and fecundity selection (Tarwater and Beissinger 2013). We found fecundity selection for early flowering, which is consistent with a large‐scale meta‐analysis of phenotypic selection on flowering phenology across systems (Munguía‐Rosas et al. 2011). Nevertheless, local trait values are much closer to the optimum from viability than fecundity selection for the 2011 cohort (Fig. 2C) and lie near the intersection of viability and fecundity selection gradients in the high elevation environment (Fig. 2I). Therefore, neglecting to account for viability selection could lead to inaccurate conclusions that populations are maladapted with trait values that fall far from fitness optima. When the action of selection via different fitness components is not additive or reinforcing, reconciling patterns of natural selection with evidence of adaptation will require evaluating selection at multiple life‐history stages (Mojica and Kelly 2010; Tarwater and Beissinger 2013).

## Conclusions

Our results illustrate that accounting for variation in trait expression and fitness across space, time, and ontogeny can improve characterizations of genetically based clines and natural selection. In our system, adaptive genetically based clines were stable across cohorts and gardens, but they were not apparent in all years. This context‐dependency indicates that temporal variation in conditions can obscure genetic clines, which may limit the ability of short‐term field studies, or experiments conducted under laboratory conditions, to detect important clines in ecologically relevant traits. Furthermore, stabilizing selection was most prominent after integrating fitness and trait data across years, suggesting that data derived from single‐year studies may not capture cumulative patterns of selection. Finally, viability selection analyses revealed that failing to account for the "invisible fraction" could lead to an incomplete view of selection acting on both juvenile and adult traits.

## Supporting information


**Fig. S1**: Genetically‐based clines in four functional traits measured in two gardens across two growing seasons for the 2012 cohort.
**Fig. S2**: Spatio‐temporal plasticity in functional traits for the 2011 and 2012 cohorts: (a) Carbon isotope discrimination (integrated water‐use efficiency, WUE), (b) specifc leaf area, (c) height at first fowering, and (d) flowering phenology (number of days elapsed between snowmelt and first flowering).

**Fig. S3**: Patterns of viability and fecundity selection (left column) and the resulting cumulative selection (right column) on (A,B) water‐use efficiency (WUE) in the 2011 cohort, (C,D) SLA in the 2012 cohort from the low‐elevation garden, (E,F) WUE in the 2012 cohort from the low‐elevation garden, (G,H) flowering phenology from the 2012 cohort in the low‐elevation garden, (I,J) WUE from the 2012 cohort in the high‐elevation garden, and (K,L) height at flowering in the 2012 cohort in the low‐elevation garden.

**Table S1**: Coordinates for populations of families included in the common garden experiment.
**Table S2**: Regression relationship used to estimate ordinal day of first flowering for the subset of plants that flowered between censuses.
**Table S3**: (A) Broad sense heritabilities for functional traits and fitness components measured in different cohorts and gardens, (B) samples sizes for traits in each season, and (C) correlations among functional traits.
**Table S4**:  Repeated measures multivariate regression results for both cohorts testing multivariate hypothesis about the stability of genetic clines across space and time, and plasticity in four phenotypes
**Table S5**: Slopes of genetically‐based clines for both cohorts (parts A and B)
**Table S6**: Accounting for neutral population genetic structure and genetically‐based clines by analyzing individual level data
**Table S7**: Aster Models and complementary analyses in SAS
**Table S8**: Direct viability, fecundity, and cumulative selection gradients derived from Aster and SAS models for the 2011 cohort.
**Table S9**: Direct, viability, fecundity, and cumulative selection gradients derived from Aster and SAS models for the 2012 cohort in both gardens.
**Table S10**: Log‐likelihood ratio tests for the significance of linear and quadratic selection gradients derived from Aster models for the 2012 cohort.
**Table S11**: SAS direct selection gradients of individual seasons for the 2011 cohort.
**Table S12**: SAS direct selection gradients of individual seasons for the 2012 cohort.
