# Supplementary material for: Integrating viability and fecundity selection to illuminate the adaptive nature of genetic clines
Source: Evol Lett. 2017 May 3;1(1):26–39. doi: 10.1002/evl3.3 (PMC6121800; doi:10.1002/evl3.3)
Supplement: Supplementary file 1 — Fig. S1: Genetically‐based clines in four functional traits measured in two gardens across two growing seasons for the 2012 cohort. Fig. S2: Spatio‐temporal plasticity in functional traits for the 2011 and 2012 cohorts: (a) Carbon isotope discrimination (integrated water‐use efficiency, WUE), (b) specifc leaf area, (c) height at first fowering, and (d) flowering phenology (number of days elapsed between snowmelt and first flowering). [file EVL3-1-26-s001.pdf]

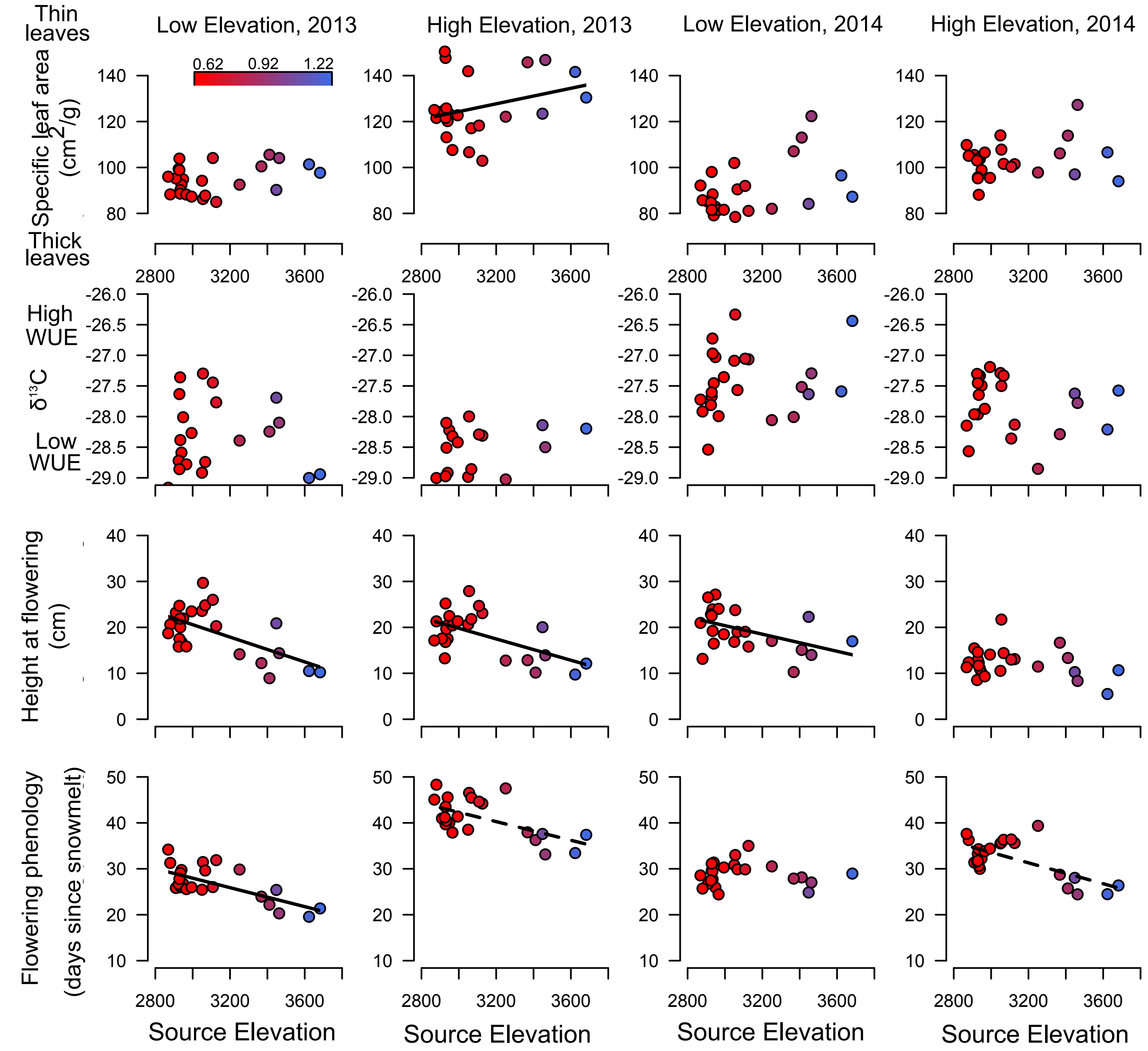

Fig. S1. Genetically-based clines in four functional traits measured in two gardens across two growing seasons for the 2012 cohort. Traits include: specific leaf area (top panels), Stable Carbon isotopes (second set of panels), height at flowering (third set of panels) and timing of first flowering (number of days elapsed since snowmelt, bottom set of panels). Data points represent family-level averages for these heritable traits. Lines represent the shape of the relationship between source elevation and trait values (only shown for significant relationships after correction for multiple tests). Panels that do not display regression lines showed no evidence of genetic clines. The color coding represents calculated Aridity Index based on climatic data extracted from WorldClim, ranging from arid low elevation populations (red) to less arid high elevation populations (blue).

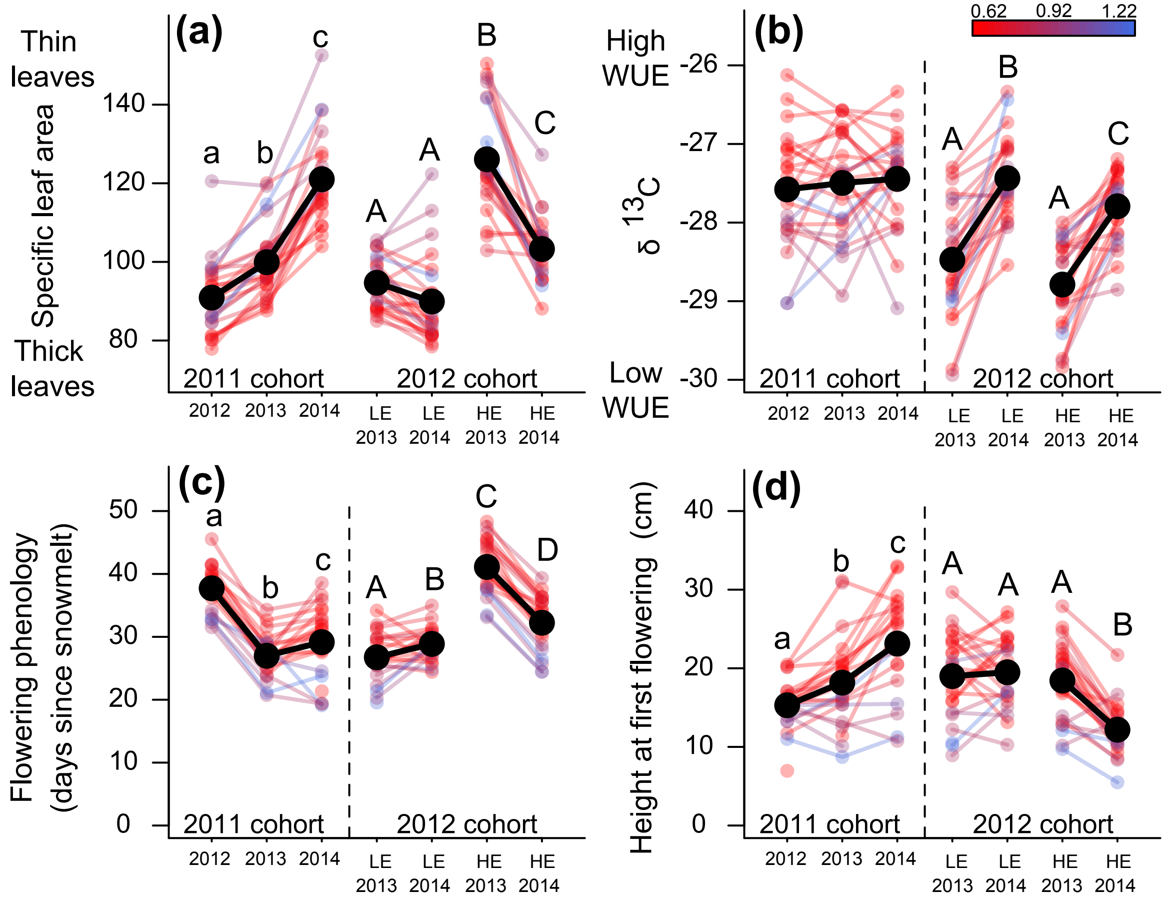

Fig. S2: Spatio-temporal plasticity in functional traits for the 2011 and 2012 cohorts: (a) Carbon isotope discrimination (integrated water-use eficiency, WUE), (b) specifc leaf area, (c) height at first fowering, and (d) fowering phenology (number of days elapsed between snowmelt and first flower). The X-axis indicates the environment,which is 3 years in the low elevation garden for the 2011 cohort (2012-2014), and two years (2013 + 2014) in each garden for the 2012 cohort (LE= low elevation; HE=high elevation). Grey circles and lines reflect norms of reaction for individual families, whereas black circles and lines indicate mean trait values across the 24 families. For the 2011 cohort, different lower case letters indicate statistically different trait values across years. For the 2012 cohort, different upper case letters indicate statistically different trait values across gardens and years. The color coding represents calculated Aridity Index based on climatic data extracted from WorldClim, ranging from arid low elevation populations (red) to less arid high elevation populations (blue).

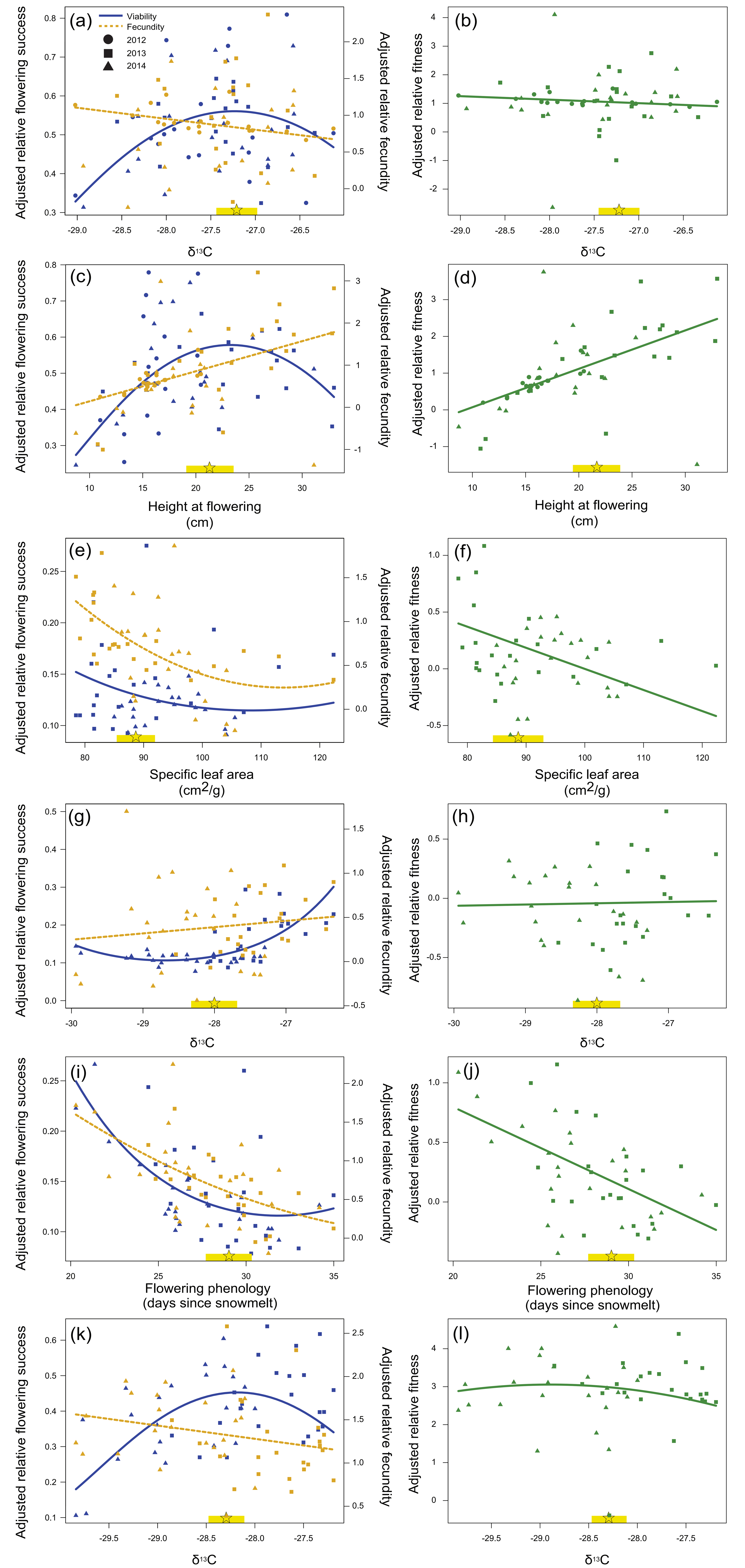

Patterns of viability and fecundity selection (left column) and the resulting cumulative selection (right column) on (a,b) WUE in the 2011 cohort, (c,d) height at flowering in the 2011 cohort, (e, f) SLA in the 2012 cohort from the low-elevation garden, (g, h) WUE in the 2012 cohort from the low-elevation garden, (i, j) flowering phenology from the 2012 cohort in the low-elevation garden, and (k, l) WUE from the 2012 cohort in the high-elevation garden. These analyses include data from all years of the study. Y-axes display adjusted fitness values, which were statistically corrected for other variables included in the models by adding residuals from full models to predicted fitness values estimated from regression coefficients for the trait of interest. We depict fitness curves using linear and (undoubled) quadratic regression coefficients from multivariate models, but we doubled quadratic regression coefficients to calculate nonlinear selection gradients in Table S8 and S10 (Stinchcombe et al. 2008). Mean trait values for local genotypes are displayed with a yellow star and are bracketed in yellow by 2*SE. Local values were extracted from models of genetically based clines (Fig. 1 and S1) for families with a source elevation of 2891m (the elevation of the lower garden) or 3133m (the elevationof the higher garden).

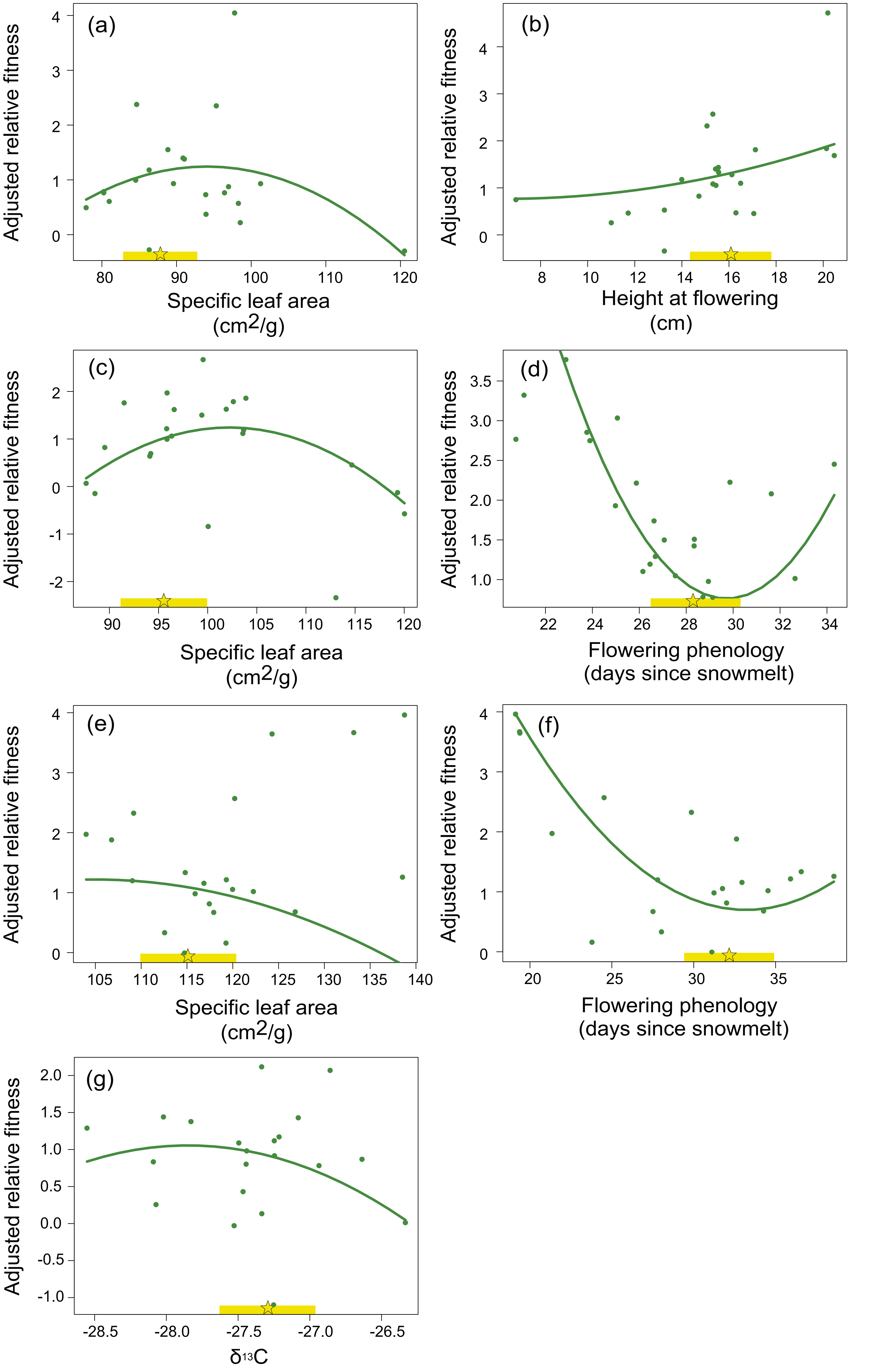

Patterns of significant quadratic cumulative selection from analyses of the 2011 cohort for each season separately on (a) SLA in 2012, (b) height at flowering in 2012, (c) SLA in 2013, (d) flowering phenology in 2013, (e) SLA in 2014, (f) flowring phenolgy in 2014, and (g) WUE in 2014. Y-axes display adjusted fitness values, which were statistically corrected for other variables included in the models by adding residuals from full models to predicted fitness values estimated from regression coefficients for the trait of interest. We depict fitness curves using linear and (undoubled) quadratic regression coefficients from multivariate models, but we doubled quadratic regression coefficients to calculate nonlinear selection gradients in Table S12 (Stinchcombe et al. 2008). Mean trait values for local genotypes are displayed with a yellow star and are bracketed in yellow by 2*SE. Local values were extracted from models of genetically based clines (Fig. 1 and S1) for families with a source elevation of 2891m (the elevation of the lower garden) or 3133m (the elevation of the higher garden).
