# Supplementary material for: Integrating viability and fecundity selection to illuminate the adaptive nature of genetic clines
Source: Evol Lett. 2017 May 3;1(1):26–39. doi: 10.1002/evl3.3 (PMC6121800; doi:10.1002/evl3.3)
Supplement: Supplementary file 2 — Fig. S3: Patterns of viability and fecundity selection (left column) and the resulting cumulative selection (right column) on (A,B) water‐use efficiency (WUE) in the 2011 cohort, (C,D) SLA in the 2012 cohort from the low‐elevation garden, (E,F) WUE in the 2012 cohort from the low‐elevation garden, (G,H) flowering phenology from the 2012 cohort in the low‐elevation garden, (I,J) WUE from the 2012 cohort in the high‐elevation garden, and (K,L) height at flowering in the 2012 cohort in the low‐elevation garden. [file EVL3-1-26-s003.pdf]

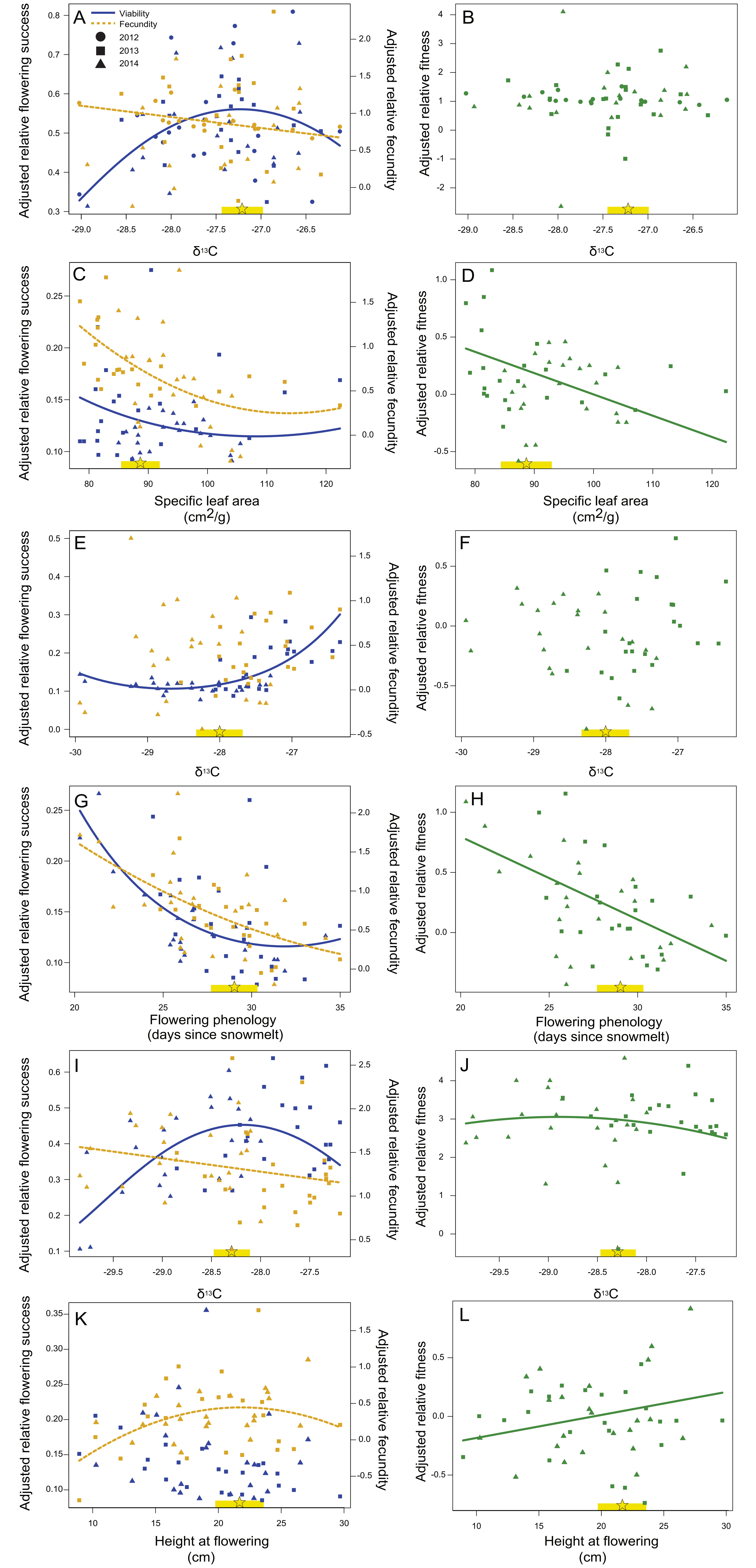

Fig. S3 Patterns of viability and fecundity selection (left column) and the resulting cumulative selection (right column) on (A,B) WUE in the 2011 cohort, (C,D) SLA in the 2012 cohort from the low-elevation garden, (E,F) WUE in the 2012 cohort from the low-elevation garden, (G,H) flowering phenology from the 2012 cohort in the low-elevation garden, (I,J) WUE from the 2012 cohort in the high-elevation garden, and (K,L) height at flowering in the 2012 cohort in the low-elevation garden. These analyses include data from all years of the study. Y-axes display adjusted fitness values, which were statistically corrected for other variables included in the models by adding residuals from full models to predicted fitness values estimated from regression coefficients for the trait of interest. We depict fitness curves using linear and (undoubled) quadratic regression coefficients from multivariate models, but we doubled quadratic regression coefficients to calculate nonlinear selection gradients in Table S8 and S10 (Stinchcombe et al. 2008). We treat results where the significance of selection coefficients derived from Aster and SAS differ as tentative. We do not include fitness curves for tentative or non-significant results. Mean trait values for local genotypes are displayed with a yellow star and are bracketed in yellow by 2*SE. Local values were extracted from models of genetically based clines (Fig. 1 and S1) for families with a source elevation of 2891m (the elevation of the lower garden) or 3133m (the elevation of the higher garden).
