# Supplementary material for: Integrating viability and fecundity selection to illuminate the adaptive nature of genetic clines
Source: Evol Lett. 2017 May 3;1(1):26–39. doi: 10.1002/evl3.3 (PMC6121800; doi:10.1002/evl3.3)
Supplement: Supplementary file 3 — Table S1: Coordinates for populations of families included in the common garden experiment. Table S2: Regression relationship used to estimate ordinal day of first flowering for the subset of plants that flowered between censuses. Table S3: (A) Broad sense heritabilities for functional traits and fitness components measured in different cohorts and gardens, (B) samples sizes for traits in each season, and (C) correlations among functional traits. Table S4: Repeated measures multivariate regression results for both cohorts testing multivariate hypothesis about the stability of genetic clines across space and time, and plasticity in four phenotypes Table S5: Slopes of genetically‐based clines for both cohorts (parts A and B) Table S6: Accounting for neutral population genetic structure and genetically‐based clines by analyzing individual level data Table S7: Aster Models and complementary analyses in SAS Table S8: Direct viability, fecundity, and cumulative selection gradients derived from Aster and SAS models for the 2011 cohort. Table S9: Direct, viability, fecundity, and cumulative selection gradients derived from Aster and SAS models for the 2012 cohort in both gardens. Table S10: Log‐likelihood ratio tests for the significance of linear and quadratic selection gradients derived from Aster models for the 2012 cohort. Table S11: SAS direct selection gradients of individual seasons for the 2011 cohort. Table S12: SAS direct selection gradients of individual seasons for the 2012 cohort. [file EVL3-1-26-s002.docx]

**Title:** Integrating temporal viability and fecundity selection to illuminate the adaptive nature of genetic clines

**Authors:** Susana M. Wadgymar, S. Caroline Daws, and Jill T. Anderson

**Supporting Tables and Documentation**

**Table of Contents:**

Table S1: Population information…………………………………………………...……………2

Table S2: Calculations of ordinal date of fowering………………………………………………3

Table S3: (A) Heritabilities……………………………………………………………………….4

(B) Sample sizes………………………………………………………………………5

(C) Correlations between traits………………………………………………………..7

Table S4: (A) Stability of genetic clines and plasticity…………………………………………..8

(B) Spatio-temporal plasticity…………………………………………………………9

Table S5: (A) Slopes of genetic clines for 2011 cohort…………………………………………10

(B) Slopes of genetic clines for 2012 cohort………………………………...….…...11

Table S6: Individual-level genetically based clines…………………………………………….12

(A) With genetic PCA………………………………………………………………..13

(B) Without genetic PCA…………………………………………………………….16

Table S7: Description of Aster models and complimentary SAS analyses…...…………………19

Table S8: Aster and SAS direct selection gradients for 2011 cohort…………………………...20

Table S9: Aster and SAS direct selection gradients for 2012 cohort …………………………..22

Table S10: Loglikelihood tests of traits and interactions for 2012 cohort ……...…….………...23

Table S11: SAS direct selection gradients for analyes of individual seasons for 2011 cohort

Cumulative selection……………………………………………………………….24

Table S12: SAS direct selection gradients for analyses of individual seasons for 2012 cohort

Cumulative selection……………………………………………………………….25

Literature cited………………………………………………………………………………….26

**Table S1: Coordinates for populations of families included in the common garden experiment.** We included one family per population.

| Family | Latitude | Longitude | Elevation (m above sea level) |
| --- | --- | --- | --- |
| GV048 | 38 58.929 | -107 00.078 | 2949 |
| GV049 | 38 59.052 | -107 00.232 | 2940 |
| GV050 | 38 58.770 | -107 00.030 | 2934 |
| GV066 | 38 58.778 | -107 00.243 | 2929 |
| GV068 | 38 57.654 | -106 59.561 | 2910 |
| GV072 | 38 58.070 | -106 59.799 | 2933 |
| GV086 | 38 57.329 | -106 59.308 | 2880 |
| GV089 | 38 56.992 | -106 59.408 | 2869 |
| GV092 | 38 57.250 | -106 59.612 | 2925 |
| GV095 | 38 57.730 | -106 59.300 | 2966 |
| GV096 | 38 57.750 | -106 59.140 | 2994 |
| GV101 | 38 58.490 | -106 59.470 | 3050 |
| GV105 | 38 58.280 | -106 59.770 | 2929 |
| GV161 | 38 59.956 | -107 01.265 | 3055 |
| GV175 | 39 00.841 | -107 02.675 | 3251 |
| GV185 | 39 00.683 | -107 02.037 | 3411 |
| GV187 | 38 59.730 | -107 01.340 | 3067 |
| GV188 | 38 59.665 | -107 01.456 | 3126 |
| GV194 | 38 59.436 | -107 01.180 | 3108 |
| GV197 | 38 59.154 | -106 58.227 | 3682 |
| GV198 | 39 00.775 | -106 56.781 | 3623 |
| GV200 | 38 59.887 | -106 58.872 | 3448 |
| GV202 | 38 58.129 | -107 01.660 | 3368 |
| GV205 | 38 58.433 | -107 02.193 | 3463 |
| Low elevation common garden | 39 02.346 | -107 03.818 | 2891 |
| High elevation common garden | 38 57.113 | -106 59.991 | 3133 |

**Table S2: Regression relationship used to estimate ordinal day of first flowering for the subset of plants that flowered between censuses**. We recorded data on phenological status, numbers of flowers, numbers of fruit, and the length of the longest fruit for each plant 3-4 times a week; in several instances, plants produced their first flower between censuses. To generate this regression relationship, we analyzed data from N=154 plants for which we have data on the exact ordinal day of first flowering and growth rate data from that day until the next census. We base this estimation on the rate of production of fruits and the rate of fruit elongation.

| Variables in the regression | |
| --- | --- |
| Code | Description |
| Elapsed days | Number of days between the ordinal day when a plant first flowered and the following census |
| LF | Length of the longest fruit (silique) on the first census after flowering |
| NF | Number of fruits (siliques) on the first census after flowering |

Regression model

Elapsed days = 0.52 +(0.54 × LF) - (0.006 × LF^2^) +(0.985 ×NF)-(0.0296×NF^2^)

Type 3 tests of fixed effects. The intercept had a parameter estimate of 0.52 ± 0.91

| Effect | F_1,149_ | p-value | parameter estimate | SE |
| --- | --- | --- | --- | --- |
| LF | 33.57 | <0.0001 | 0.5377 | 0.09281 |
| LF^2^ | 17.45 | <0.0001 | -0.00632 | 0.001514 |
| NF | 11.74 | 0.0008 | 0.9825 | 0.2867 |
| NF^2^ | 6.26 | 0.0134 | -0.0296 | 0.01182 |

We then took this regression model and applied it to all plants to adjust the timing of first flowering. Plants that flowered on the day of a census would have had 0 fruits on that day, which means that their ordinal day of flower flowering would not change. This calculation only affects plants that had both flowers and fruits on the census day when flowering was first noted.

| Code | Description |
| --- | --- |
| DFF | Calculated day of first flowering |
| OD | Ordinal day of year when a flower was first recorded on a plant (census when flowering was first noted) |

DFF = OD - Elapsed days

**Table S3: (A) Broad sense heritabilities for functional traits and fitness components measured in different cohorts and gardens, (B) samples sizes for traits in each season, and (C) correlations among functional traits.** We used data from all growing seasons to estimate heritabilities. We present raw p-values (based on likelihood ratio tests with and without the random effect of family) as well as false discovery rate corrected p-values based on the Benjamini-Hochberg procedure ([1995](#_ENREF_2)). We have highlighted in bold the significant corrected p-values. Under α=0.05, we would have expected 0.9 signifcant heritabilities in our 18 separate tests; instead we found 18 significant heritabilities after applying the Benjamini-Hochberg procedure.

| Trait | Garden | Cohort | Growing season | H^2^ | χ^2^ | raw p-value | FDR corrected p-value |
| --- | --- | --- | --- | --- | --- | --- | --- |
| Specific leaf area | low elevation | 2011 | 2012-2014 | 0.083± 0.022 | 45.2 | **<0.0001** | **<0.0001** |
| Water-use efficiency (δ^13^C) | low elevation | 2011 | 2012-2014 | 0.291± 0.076 | 18 | **<0.0001** | **<0.0001** |
| Flowering phenology | low elevation | 2011 | 2012-2014 | 0.376± 0.057 | 164.3 | **<0.0001** | **<0.0001** |
| height at flowering | low elevation | 2011 | 2012-2014 | 0.255± 0.058 | 69.2 | **<0.0001** | **<0.0001** |
| Flowering success | low elevation | 2011 | 2012-2014 | 0.362± 0.054 | 272.3 | **<0.0001** | **<0.0001** |
| Fecundity, among plants that flowered | low elevation | 2011 | 2012-2014 | 0.13± 0.039 | 28.1 | **<0.0001** | **<0.0001** |
| Specific leaf area | low elevation | 2012 | 2013-2014 | 0.209± 0.045 | 102 | **<0.0001** | **<0.0001** |
| Water-use efficiency (δ^13^C) | low elevation | 2012 | 2013-2014 | 0.202± 0.072 | 13.8 | **0.0002** | **0.0006** |
| Flowering phenology | low elevation | 2012 | 2013-2014 | 0.408± 0.069 | 78.5 | **<0.0001** | **<0.0001** |
| height at flowering | low elevation | 2012 | 2013-2014 | 0.431± 0.072 | 72.3 | **<0.0001** | **<0.0001** |
| Flowering success | low elevation | 2012 | 2013-2014 | 0.139± 0.047 | 22.2 | **<0.0001** | **<0.0001** |
| Fecundity, among plants that flowered | low elevation | 2012 | 2013-2014 | 0.124± 0.052 | 11.2 | **0.00082** | **0.0008** |
| Specific leaf area | high elevation | 2014 | 2013-2014 | 0.214± 0.045 | 142.9 | **<0.0001** | **<0.0001** |
| Water-use efficiency (δ^13^C) | high elevation | 2012 | 2013-2014 | 0.22± 0.078 | 11.9 | **0.00056** | **0.0008** |
| Flowering phenology | high elevation | 2012 | 2013-2014 | 0.236± 0.048 | 218.3 | **<0.0001** | **<0.0001** |
| height at flowering | high elevation | 2012 | 2013-2014 | 0.411± 0.057 | 507 | **<0.0001** | **<0.0001** |
| Flowering success | high elevation | 2012 | 2013-2014 | 0.349± 0.062 | 141.8 | **<0.0001** | **<0.0001** |
| Fecundity, among plants that flowered | high elevation | 2012 | 2013-2014 | 0.162± 0.042 | 109.9 | **<0.0001** | **<0.0001** |

**(B): Sample sizes for each trait in each cohort, garden, and season.**

| Trait | Cohort | Garden | Season | Number of individuals sampled |
| --- | --- | --- | --- | --- |
| Specific leaf area | 2011 | low elevation | 2012 | 488 |
| Water-use efficiency (δ^13^C) | 2011 | low elevation | 2012 | 77^(see Note 1)^ |
| Height at flowering | 2011 | low elevation | 2012 | 316 |
| Flowering phenology | 2011 | low elevation | 2012 | 319 |
| Specific leaf area | 2011 | low elevation | 2013 | 543 |
| Water-use efficiency (δ^13^C) | 2011 | low elevation | 2013 | 77 ^(see Note 1)^ |
| Height at flowering | 2011 | low elevation | 2013 | 164 |
| Flowering phenology | 2011 | low elevation | 2013 | 164 |
| Specific leaf area | 2011 | low elevation | 2014 | 380 |
| Water-use efficiency (δ^13^C) | 2011 | low elevation | 2014 | 74 ^(see Note 1)^ |
| Height at flowering | 2011 | low elevation | 2014 | 162 |
| Flowering phenology | 2011 | low elevation | 2014 | 161 |
| Specific leaf area | 2012 | low elevation | 2013 | 365 |
| Water-use efficiency (δ^13^C) | 2012 | low elevation | 2013 | 89 ^(see Note 2)^ |
| Height at flowering | 2012 | low elevation | 2013 | 131 |
| Flowering phenology | 2012 | low elevation | 2013 | 132 |
| Specific leaf area | 2012 | low elevation | 2014 | 520 |
| Water-use efficiency (δ^13^C) | 2012 | low elevation | 2014 | 71 ^(see Note 2)^ |
| Height at flowering | 2012 | low elevation | 2014 | 127 |
| Flowering phenology | 2012 | low elevation | 2014 | 127 |
| Specific leaf area | 2012 | high elevation | 2013 | 431 |
| Water-use efficiency (δ^13^C) | 2012 | high elevation | 2013 | 110 ^(see Note 2)^ |
| Height at flowering | 2012 | high elevation | 2013 | 836 |
| Flowering phenology | 2012 | high elevation | 2013 | 840 |
| Specific leaf area | 2012 | high elevation | 2014 | 438 |
| Water-use efficiency (δ^13^C) | 2012 | high elevation | 2014 | 67 ^(see Note 2)^ |
| Height at flowering | 2012 | high elevation | 2014 | 136 |
| Flowering phenology | 2012 | high elevation | 2014 | 136 |

Notes:

^1^ For water-use efficiency (δ^13^C), in the 2011 cohort, we sampled N=79 individual plants (2.9 ± 0.34 individuals/ family; mean ±SD, 24 families), of which 72 individuals were sampled in all three growing years (3.0 ± 0.78 individuals/family, 23 families), leaves of 6 individuals were only available for two growing years, and leaves of one individual were available for one year only, for a total of 229 records across all three years (number of individuals ×number of years sampled). We were unable to sample siblings of one high elevation family (3463m) across all three years in the low elevation garden (2890m), but we included leaves of three individuals in two years and one individual in one year.

^2^ For water-use efficiency (δ^13^C) in the 2012 cohort, we sampled 90 individual plants in the low elevation garden (3.75 ± 1.11 individuals/ family; mean ±SD, 24 families), of which 70 individuals were sampled in both 2013 and 2014 (2.92 ± 0.71 individuals/family, 24 families), and leaves of 20 individuals were available for one year only, for a total of 160 records across 2013 and 2014 (number of individuals ×number of years sampled). In the high elevation garden, we sampled a total of 112 individual plants (4.67 ± 1.55 individuals/ family; mean ±SD, 23 families), of which 66 individuals were sampled in both 2013 and 2014 (2.75 ± 0.74 individuals/family, 23 families), and leaves of 45 individuals were available for one year only, for a total of 177 records across 2013 and 2014 (number of individuals ×number of years sampled). One high elevation family (3411m) did not have sufficient survival to sample even one individual plant in one growing year in the high elevation garden.

Number of leaves collected per trait for foliar traits (SLA + δ^13^C)

| Cohort | Garden | Season | # leaves per plant (mean ± SD) |
| --- | --- | --- | --- |
| 2011 | low elevation | 2012 | 1.82 ± 0.92 |
| 2011 | low elevation | 2013 | 4.77 ± 1.29 |
| 2011 | low elevation | 2014 | 3.06 ± 0.40 |
| 2012 | low elevation | 2013 | 2.27 ± 0.62 |
| 2012 | low elevation | 2014 | 2.92 ± 0.32 |
| 2012 | high elevation | 2013 | 2.58 ± 0.75 |
| 2012 | high elevation | 2014 | 3.19 ± 1.08 |

**(C): Pearson Correlation coefficients (r) among functional traits across the entire dataset, using individual-level data**. Under each correlation coefficient is the p-value (bolded in cases of statistical significance) and sample sizes (N) for the correlation. We did not apply a correction for multiple tests in this Table.

|  |  | δ^13^C | Specific leaf area | Flowering phenology | Height at flowering |
| --- | --- | --- | --- | --- | --- |
| r | δ^13^C | 1 |  |  |  |
| p-value |  |  |  |  |  |
| N |  | 565 |  |  |  |
| r | Specific leaf area | **-0.28** | 1 |  |  |
| p-value |  | **<0.0001** |  |  |  |
| N |  | **564** | 3165 |  |  |
| r | Flowering phenology | **-0.276** | **0.20** | 1 |  |
| p-value |  | **<0.0001** | **<0.0001** |  |  |
| N |  | **207** | **950** | 1879 |  |
| r | Height at flowering | -0.062 | 0.039 | **-0.059** | 1 |
| p-value |  | 0.37 | 0.23 | **0.0112** |  |
| N |  | 207 | 948 | **1871** | 1872 |

**Table S4:  Repeated measures multivariate regression results for both cohorts testing multivariate hypothesis about the stability of genetic clines across space and time, and plasticity in four phenotypes** (flowering time, height at flowering, specific leaf area, and integrated water-use efficiency as measured through stable Carbon isotopes). These models simultaneously evaluate all four traits and their interactions with source elevation, growing season, and (for the 2012 cohort) experimental garden. Significant interactions between phenotype and other explanatory variables indicate that effects of elevation, season, garden and their interactions differ by trait. We used preplanned contrast and estimate statements to examine clines and plasticity separately for each trait. We planted the 2011 cohort into the lower elevation garden and monitored phenotypes for 3 growing seasons. The following year (2012 cohort), we planted full siblings from the same 24 maternal families as in the 2011 cohort into both high and low elevation gardens, and monitored phenotypes for two growing seasons.

| Effect | 2011 cohort | | 2012 cohort | |
| --- | --- | --- | --- | --- |
|  | F-value | p-value | F-value | p-value |
| Phenotype (P) | F_4,37.6_=13.02 | **<0.0001** | F_4,66.7_=12.6 | **<0.0001** |
| P × Elevation | F_4,37.6_=13.12 | **<0.0001** | F_4,66.9_=12.67 | **<0.0001** |
| P × Season | F_8,66.6_=3.65 | **0.0014** | F_4,67.3_=4.89 | **0.0016** |
| P × Elevation × Season | F_8,66.7_=2.84 | **0.009** | F_4,67.5_=2.65 | **0.0404** |
| P × Garden | NA | NA | F_4,66.9_=2.93 | **0.027** |
| P × Elevation × Garden | NA | NA | F_5,66.9_=1.21 | 0.315 |
| P × Season × Garden | NA | NA | F_4,67.3_=2.86 | **0.0301** |
| P × Elevation × Season × Garden | NA | NA | F_5,67.5_=4.68 | **0.0022** |

**(B) Patterns of spatiotemporal plasticity:** We have adjusted all p-values for contrasts across sites and gardens using the Benjamini-Hochberg procedure. Spatial plasticity was co-gradient with genetic clines for three traits (Fig. S2): SLA [greater trait values in the higher vs. lower garden (2013: t_83.3_=-11.2, p<0.0001; 2014: t_82.6_=-4.74, p<0.0001)], δ^13^C [WUE was greater in the low vs. high elevation garden in 2014 (t_83.7_=2.12, p=0.0497) and marginally so in 2013 (t_83.6_=1.91, p=0.068)], and height at flowering [smaller size at flowering in the higher garden in 2014 (t_82.8_=6.14, p<0.0001), but no pattern in 2013 (t_82.2_=0.5, p=0.62)]. Counter to expectations, in both years, plants flowered earlier (relative to snowmelt) in the lower garden than their siblings did in the higher garden (2013: t_86.5_=-17.2, p<0.0001; 2014: t_86.8_=-4.1, p=0.0002; Fig. S2d).

*Temporal plasticity*: We found no temporal plasticity in δ^13^C in the 2011 cohort (F_2,61.9_=0.36, p=0.70). However, the significant year by garden interaction for the 2012 cohort (F_3,75.5_= 41.4, p<0.0001) indicated plasticity in integrated WUE (Fig. S2a). Contrary to predictions, plants in both gardens had higher WUE in the benign year (2014) compared to the drier 2013 (lower garden: t_66_=-7.92, p<0.0001; higher garden: t_66.5_=-7.49, p<0.0001), perhaps reflecting differential trait expression across ontogeny. Both cohorts exhibited temporal plasticity in specific leaf area (Fig. S2b). For the 2011 cohort, temporal plasticity was concordant with expectations (F_2,53.4_=114.7, p<0.0001, Fig. S2c): increasing from the driest year (2012) to 2013 (t_52.3_=-5.34, p<0.0001) and from 2013 to 2014 (t_52.3_=-12.5, p<0.0001). For the 2012 cohort, a significant season by garden interaction (F_3,77.1_=67.97, p<0.0001) revealed countergradient temporal plasticity in the both gardens, with lower SLA values during 2014 relative to 2013 (low elevation: t_68.7_=2.19, p=0.037; high elevation: t_69.7_=10.51, p<0.0001). Temporal plasticity in height at flowering was concordant with predictions for the 2011 cohort (F_2,48.6_=24.34, p<0.0001, Fig. S2d). Plants were significantly shorter at flowering in 2012 than in the following years (2012 vs. 2013: t_47.5_=-2.88, p=0.0089; 2012 vs. 2014: t_55.4_=-6.8, p<0.0001), and in 2013 vs. 2014 (t_47.8_=-5.49, p<0.0001). However, in the 2012 cohort, temporal plasticity occurred in the opposite direction (garden × season: F_3,78.2_=21.9, p<0.0001, Fig. S2d): Plants were shorter at flowering in 2014 than 2013 in the higher garden (t_70.7_=7.06, p<0.0001), but not in the lower garden (t_71.8_=-0.28, p=0.78). Finally, for the 2011 cohort, a significant effect of year (F_2,53.2_=96.2, p<0.0001) revealed earlier flowering in 2012 vs. 2013 (t_52.4_=13.85, p<0.0001) and 2014 (t_58.4_=9.06, p<0.0001), but slightly earlier flowering in 2013 than 2014 (t_52.8_=-2.50, p=0.021; Fig. 2e). A garden by season interaction for the 2012 cohort (F_3,73_=119.3, p<0.0001) arose because flowering was significantly delayed in 2013 relative to 2014 in the higher garden (t_60.9_=13.7, p<0.0001), but was earlier in 2013 than 2014 in the lower garden (t_61.7_=-3.11, p=0.0038; Fig. 2e).

Whereas spatial plasticity matched expectations, temporal plasticity was often discordant with clines. Temporal plasticity in δ^13^C could reflect improved water acquisition in larger plants, whereas counter-gradient plasticity in other traits may indicate environmental constraints on trait expression.

**Table S5: Slopes of genetically-based clines for both cohorts (parts A and B)**

(A) Slopes of genetic clines in traits across three growing seasons for the 2011 cohort based on multivariate repeated measures regression. Traits were standardized to a mean of 0 and standard deviation before analysis. Slopes (β) represent changes in standardized trait values per 1km gain in source elevation. Under α=0.05, we would have expected 0.6 signifcant tests; instead we found 8 signifcant clines (of 12 tests) after correction for multiple testing using the Benjamini-Hochberg procedure. We show raw and corrected p-values. Flowering time was measured as elapsed days since snowmelt.

| Phenotype | Season | β ± SE | degrees of freedom | t-value | raw p-value | FDR p-value |
| --- | --- | --- | --- | --- | --- | --- |
| Specific leaf area (cm^2^/g) | 2012 | 0.846± 0.44 | 61.2 | 1.92 | 0.06 | 0.08 |
| Specific leaf area (cm^2^/g) | 2013 | 1.208± 0.44 | 61.2 | 2.74 | 0.008 | **0.014** |
| Specific leaf area (cm^2^/g) | 2014 | 1.628± 0.44 | 61.2 | 3.69 | 0.00048 | **0.0019** |
| Water-use efficiency (δ^13^C) | 2012 | -2.96± 0.81 | 51.6 | -3.65 | 0.00062 | **0.0019** |
| Water-use efficiency (δ^13^C) | 2013 | -2.12± 0.81 | 51.6 | -2.62 | 0.012 | **0.017** |
| Water-use efficiency (δ^13^C) | 2014 | -1± 0.81 | 51.6 | -1.23 | 0.22 | 0.24 |
| Height at flowering (cm) | 2012 | -0.4± 0.59 | 57.1 | -0.68 | 0.497 | 0.497 |
| Height at flowering (cm) | 2013 | -1.69± 0.59 | 57 | -2.86 | 0.0059 | **0.0117** |
| Height at flowering (cm) | 2014 | -2.93± 0.61 | 58.1 | -4.79 | 0.00001 | **0.0001** |
| Flowering time (days since snowmelt) | 2012 | -1.58± 0.49 | 52.6 | -3.22 | 0.002 | **0.005** |
| Flowering time (days since snowmelt) | 2013 | -0.83± 0.49 | 52.5 | -1.7 | 0.094 | 0.113 |
| Flowering time (days since snowmelt) | 2014 | -2.2± 0.51 | 53.9 | -4.34 | 0.00006 | **0.0004** |

**(B)** Slopes of genetic clines in traits across two growing seasons for the 2012 cohort planted into low and high elevation common gardens, based on multivariate repeated measures regression. Traits were standardized to a mean of 0 and standard deviation before analysis. Slopes represent changes in standardized trait values per 1km gain in source elevation. Under α=0.05, we would have expected 0.8 signifcant tests, instead we found 7 signifcant clines (of 16 tests) after correction for multiple testing using the Benjamini-Hochberg procedure. We show both raw and corrected p-values.

| Trait | Garden | Season | β ± SE | Degrees of freedom | t-value | raw p-value | FDR p-value |
| --- | --- | --- | --- | --- | --- | --- | --- |
| Specific leaf area (cm^2^/g) | low elevation | 2013 | 0.057± 0.05 | 82.6 | 1.18 | 0.24 | 0.43 |
| Specific leaf area (cm^2^/g) | low elevation | 2014 | 0.127± 0.05 | 82.6 | 2.63 | 0.01 | **0.03** |
| Specific leaf area (cm^2^/g) | high elevation | 2013 | 0.111± 0.05 | 84.5 | 2.25 | 0.03 | *0.05* |
| Specific leaf area (cm^2^/g) | high elevation | 2014 | 0.038± 0.05 | 82.6 | 0.78 | 0.44 | 0.6 |
| Water-use efficiency (δ^13^C) | low elevation | 2013 | -0.009± 0.06 | 83.4 | -0.15 | 0.88 | 0.88 |
| Water-use efficiency (δ^13^C) | low elevation | 2014 | 0.044± 0.06 | 83.4 | 0.7 | 0.48 | 0.6 |
| Water-use efficiency (δ^13^C) | high elevation | 2013 | 0.027± 0.06 | 84.1 | 0.42 | 0.68 | 0.77 |
| Water-use efficiency (δ^13^C) | high elevation | 2014 | -0.048± 0.06 | 84.5 | -0.75 | 0.46 | 0.6 |
| Height at flowering (cm) | low elevation | 2013 | -0.25± 0.06 | 82.2 | -4.04 | <0.0001 | **<0.0001** |
| Height at flowering (cm) | low elevation | 2014 | -0.173± 0.07 | 87.3 | -2.59 | 0.01 | **0.03** |
| Height at flowering (cm) | high elevation | 2013 | -0.215± 0.06 | 82.2 | -3.47 | <0.0001 | **<0.0001** |
| Height at flowering (cm) | high elevation | 2014 | -0.065± 0.06 | 82.2 | -1.05 | 0.29 | 0.47 |
| Flowering time (days since snowmelt) | low elevation | 2013 | -0.155± 0.04 | 86.5 | -4.24 | <0.0001 | **<0.0001** |
| Flowering time (days since snowmelt) | low elevation | 2014 | -0.014± 0.04 | 88.6 | -0.35 | 0.72 | 0.77 |
| Flowering time (days since snowmelt) | high elevation | 2013 | -0.153± 0.04 | 86.5 | -4.17 | <0.0001 | **<0.0001** |
| Flowering time (days since snowmelt) | high elevation | 2014 | -0.171± 0.04 | 86.5 | -4.67 | <0.0001 | **<0.0001** |

**Table S6: Accounting for neutral population genetic structure and genetically-based clines by analyzing individual level data**

As a first step in assessing the potential contribution of non-adaptive processes to genetic clines in our system, we leveraged data from an earlier study that genotyped a regional pool of 97 maternal families at 13 polymorphic microsatellite loci and documented moderate genetic isolation by distance ([Anderson et al. 2015](#_ENREF_1)). We included 21 of these 97 genotyped families in our common gardens. Following Kooyers et al. ([2015](#_ENREF_6)), we used the R package adegenet ([Jombart 2008](#_ENREF_5)) to conduct principal components analysis to reduce dimensionality of the 13 microsatellite loci to two principal components (genetic PC1 and PC2), which represent neutral genetic differentiation. We then included PC1 and PC2 as covariates in complementary analyses. Our statistical power is diminished because these analyses include only 21 of the 24 families in our study; therefore, we conducted multivariate repeated measures regressions using individual-level data with random effects for family and block (or block within garden). If source elevation remains a significant predictor of trait variation when genetic PC1 and PC2 are included in models, clines could be shaped or maintained by selection. We compared results of individual-level analyses with and without the genetic principal components.

To examine the role of neutral processes in structuring clines, we analyzed individual-level data with and without genetic principal components (PCs). Models of individual-level data without genetic PCs generated nearly identical results as the family-level data (Table S3). Genetic PC1 explained 11.58% of the variance in microsatellites and genetic PC2 explained 9.03% of the variance. A model with genetic PCs as fixed factors revealed significant negative relationships between three traits and source elevation for the 2011 cohort, consistent with expectations and previous analyses: δ^13^C in 2012 (t_161_=-2.65, FDR-adjusted p-value=0.027), height at flowering in 2014 (t_97.3_=-5.2, FDR-adjusted p-value<0.0001), and flowering phenology in 2012 (t_49.3_=-2.87, FDR-adjusted p-value=0.025) and 2014 (t_75.4_=-3.34, p=0.0079). In these models, we found no evidence for genetic clines in SLA. For the 2012 cohort, models with genetic PCs revealed the expected negative relationships with source elevation for flowering phenology at the lower garden in 2013 (t_46.8_=-3.51 p=0.011) and the higher garden in 2014 (t_46.1_=-3.44, p=0.011), but not for other traits.

Below, we present individual-level models: (A) with genetic PC, and (B) without genetic PC.

(A) Results of multivariate repeated measures regressions using individual-level data with genetic principal components (genetic PC1 and genetic PC2). Slopes of genetic clines in functional traits represent changes in standardized trait values per 100m gain in source elevation. Flowering time was measured as elapsed days since snowmelt and was log transformed.

| Effect | 2011 cohort | | 2012 cohort | |
| --- | --- | --- | --- | --- |
|  | F-value | p-value | F-value | p-value |
| Phenotype (P) | F_4,95.6_=3.89 | **0.0057** | F_4,123_=3.41 | **0.011** |
| P × Elevation | F_4,95.1_=4.41 | **0.0026** | F_4,123_=4.30 | **0.0027** |
| P × Season | F_8,674_=6.89 | **<0.0001** | F_4,790_=7.31 | **<0.0001** |
| P × Elevation × Season | F_8,676_=5.67 | **<0.0001** | F_4,788_=5.31 | **0.0003** |
| P × Garden | NA | NA | F_4,826_=3.10 | **0.0151** |
| P × Elevation × Garden | NA | NA | F_4,819_=1.8 | 0.11 |
| P × Season × Garden | NA | NA | F_4,790_=7.33 | **<0.0001** |
| P × Elevation × Season × Garden | NA | NA | F_4,788_=10.6 | **<0.0001** |
| P × genetic PC1 | F_4,95_=8.11 | **<0.0001** | F_4,119_=33.85 | **<0.0001** |
| P × genetic PC2 | F_4,94.5_=9.03 | **<0.0001** | F_4,118_=56.38 | **<0.0001** |
| Block | χ^2^=17.3 | **<0.0001** | χ^2^=64.6 | **<0.0001** |
| Maternal family | χ^2^=30.3 | **<0.0001** | χ^2^=207.3 | **<0.0001** |

Genetic cline estimates

| Trait | Cohort | Garden | Growing season | β ± SE | Degrees of freedom | t-value | raw p-value | FDR-adjusted p-value | Consistent with analyses (family and individual) without genetic PCs in terms of significance and direction of cline? |
| --- | --- | --- | --- | --- | --- | --- | --- | --- | --- |
| Specific leaf area | 2011 | low elevation | 2012 | -0.49± 0.334 | 33.5 | -1.46 | 0.15 | 0.23 |  |
| Specific leaf area | 2011 | low elevation | 2013 | -0.35± 0.334 | 33.5 | -1.06 | 0.3 | 0.4 | This cline is significantly positive in models that exclude genetic principal components |
| Specific leaf area | 2011 | low elevation | 2014 | -0.1± 0.352 | 41.2 | -0.28 | 0.78 | 0.78 | This cline is significantly positive in models that exclude genetic principal components |
| Water-use efficiency (δ^13^C) | 2011 | low elevation | 2012 | -1.76± 0.666 | 161 | -2.65 | 0.009 | **0.027** | yes |
| Water-use efficiency (δ^13^C) | 2011 | low elevation | 2013 | -1.19± 0.671 | 161 | -1.77 | 0.078 | 0.16 | This cline is significantly negative in models that exclude genetic principal components |
| Water-use efficiency (δ^13^C) | 2011 | low elevation | 2014 | -0.45± 0.675 | 164 | -0.67 | 0.51 | 0.58 |  |
| Height at flowering | 2011 | low elevation | 2012 | -0.25± 0.397 | 61.5 | -0.63 | 0.53 | 0.58 |  |
| Height at flowering | 2011 | low elevation | 2013 | -0.85± 0.423 | 77.1 | -2.0 | 0.049 | 0.12 | This cline is significantly negative in models that exclude genetic principal components |
| Height at flowering | 2011 | low elevation | 2014 | -2.34± 0.451 | 97.3 | -5.2 | <0.0001 | **<0.0001** | yes |
| Flowering phenology (elapsed days since snowmelt) | 2011 | low elevation | 2012 | -1.06± 0.369 | 47.7 | -2.87 | 0.0061 | **0.025** | yes |
| Flowering phenology (elapsed days since snowmelt) | 2011 | low elevation | 2013 | -0.66± 0.389 | 58.7 | -1.69 | 0.096 | 0.16 |  |
| Flowering phenology (elapsed days since snowmelt) | 2011 | low elevation | 2014 | -1.38± 0.414 | 75.4 | -3.34 | 0.0013 | **0.0079** | yes |
| Specific leaf area | 2012 | low elevation | 2013 | -0.71± 0.379 | 38.2 | -1.87 | 0.069 | 0.19 |  |
| Specific leaf area | 2012 | low elevation | 2014 | -0.4± 0.362 | 32.3 | -1.12 | 0.281 | 0.42 | This cline is significantly positive in models that exclude genetic principal components |
| Specific leaf area | 2012 | high elevation | 2013 | 0.178± 0.382 | 40.1 | 0.44 | 0.644 | 0.74 |  |
| Specific leaf area | 2012 | high elevation | 2014 | -0.66± 0.366 | 33.8 | -2.02 | 0.078 | 0.19 |  |
| Water-use efficiency (δ^13^C) | 2012 | low elevation | 2013 | 0.896± 0.581 | 148 | 1.54 | 0.125 | 0.22 |  |
| Water-use efficiency (δ^13^C) | 2012 | low elevation | 2014 | 1.191± 0.613 | 168 | 1.97 | 0.054 | 0.19 |  |
| Water-use efficiency (δ^13^C) | 2012 | high elevation | 2013 | 0.977± 0.557 | 130 | 1.75 | 0.082 | 0.19 |  |
| Water-use efficiency (δ^13^C) | 2012 | high elevation | 2014 | 0.105± 0.621 | 170 | 0.09 | 0.866 | 0.87 |  |
| Height at flowering | 2012 | low elevation | 2013 | -0.47± 0.439 | 70.1 | -1.09 | 0.286 | 0.42 | This cline is significantly negative in models that exclude genetic principal components |
| Height at flowering | 2012 | low elevation | 2014 | 0.086± 0.512 | 126 | 0.16 | 0.868 | 0.87 |  |
| Height at flowering | 2012 | high elevation | 2013 | -0.34± 0.356 | 30.4 | -0.97 | 0.352 | 0.47 | This cline is significantly negative in models that exclude genetic principal components |
| Height at flowering | 2012 | high elevation | 2014 | 0.681± 0.437 | 68.4 | 1.54 | 0.124 | 0.22 |  |
| Flowering phenology (elapsed days since snowmelt) | 2012 | low elevation | 2013 | -1.39± 0.398 | 46.8 | -3.51 | 0.001 | **0.011** | yes |
| Flowering phenology (elapsed days since snowmelt) | 2012 | low elevation | 2014 | 0.254± 0.453 | 77.5 | 0.55 | 0.577 | 0.71 |  |
| Flowering phenology (elapsed days since snowmelt) | 2012 | high elevation | 2013 | -0.64± 0.338 | 24.6 | -1.92 | 0.07 | 0.19 | This cline is significantly negative in models that exclude genetic principal components |
| Flowering phenology (elapsed days since snowmelt) | 2012 | high elevation | 2014 | -1.35± 0.396 | 46.1 | -3.44 | 0.001 | **0.011** | yes |

(B) Results of multivariate repeated measures regression analysis of individual level analysis for each cohort. Traits were standardized to a mean of 0 and standard deviation before analysis. Slopes of genetic clines represent changes in standardized trait values per 100m gain in source elevation.

| Effect | 2011 cohort | | 2012 cohort | |
| --- | --- | --- | --- | --- |
|  | F-value | p-value | F-value | p-value |
| Phenotype (P) | F_4,128_=45.33 | **<0.0001** | F_4,163_=61.87 | **<0.0001** |
| P × Elevation | F_4,129_=47.84 | **<0.0001** | F_4,163_=72.1 | **<0.0001** |
| P × Season | F_8,796_=6.46 | **<0.0001** | F_4,913_=7.05 | **0.0001** |
| P × Elevation × Season | F_8,799_=5.48 | **<0.0001** | F_4,912_=4.35 | **0.0017** |
| P × Garden | NA | NA | F_4,957_=5.03 | **0.0005** |
| P × Elevation × Garden | NA | NA | F_4,947_=2.38 | **0.0498** |
| P × Season × Garden | NA | NA | F_4,914_=7.56 | **<0.0001** |
| P × Elevation × Season × Garden | NA | NA | F_4,913_=10.63 | **<0.0001** |
| Block | χ^2^=18.0 | **<0.0001** | χ^2^=85.4 | **<0.0001** |
| Maternal family | χ^2^=43.4 | **<0.0001** | χ^2^=222.8 | **<0.0001** |

Genetic cline estimates

| Trait | Cohort | Garden | Season | β ± SE | Degrees of freedom | t-value | Raw p-value | FDR-adjusted p-value | Consistent with family-level analysis? | Concordant with expectations? |
| --- | --- | --- | --- | --- | --- | --- | --- | --- | --- | --- |
| Specific leaf area | 2011 | low elevation | 2012 | 0.39± 0.206 | 69.3 | 1.89 | 0.062 | 0.068 | yes |  |
| Specific leaf area | 2011 | low elevation | 2013 | 0.52± 0.207 | 70.8 | 2.51 | 0.014 | **0.019** | yes | yes |
| Specific leaf area | 2011 | low elevation | 2014 | 0.785± 0.238 | 118 | 3.31 | 0.00125 | **0.0021** | yes | yes |
| Water-use efficiency (δ13C) | 2011 | low elevation | 2012 | -2.04± 0.466 | 224 | -4.37 | 0.00002 | **<0.0001** | yes | yes |
| Water-use efficiency (δ13C) | 2011 | low elevation | 2013 | -1.5± 0.458 | 223 | -3.28 | 0.0012 | **0.0021** | yes | yes |
| Water-use efficiency (δ13C) | 2011 | low elevation | 2014 | -0.48± 0.477 | 225 | -1.01 | 0.31 | 0.31 | yes |  |
| Height at flowering | 2011 | low elevation | 2012 | -0.48± 0.248 | 127 | -1.94 | 0.055 | 0.066 | yes | yes |
| Height at flowering | 2011 | low elevation | 2013 | -0.9± 0.31 | 250 | -2.89 | 0.0043 | **0.0064** | yes | yes |
| Height at flowering | 2011 | low elevation | 2014 | -2.38± 0.358 | 352 | -6.64 | <0.0001 | **<0.0001** | yes | yes |
| Flowering phenology (elapsed days since snowmelt) | 2011 | low elevation | 2012 | -1.42± 0.222 | 89.4 | -6.4 | <0.0001 | **<0.0001** | yes | yes |
| Flowering phenology (elapsed days since snowmelt) | 2011 | low elevation | 2013 | -1.2± 0.271 | 178 | -4.43 | 0.00002 | **<0.0001** | This cline is not significant in the family-level analysis | yes |
| Flowering phenology (elapsed days since snowmelt) | 2011 | low elevation | 2014 | -1.82± 0.311 | 277 | -5.84 | <0.0001 | **<0.0001** | yes | yes |
| Specific leaf area | 2012 | low elevation | 2013 | 0.391± 0.246 | 107 | 1.59 | 0.12 | 0.17 | yes |  |
| Specific leaf area | 2012 | low elevation | 2014 | 0.67± 0.223 | 74.2 | 3 | 0.0037 | **0.0073** | yes | yes |
| Specific leaf area | 2012 | high elevation | 2013 | 0.809± 0.255 | 120 | 3.18 | 0.0019 | **0.0043** | This cline is marginal in the family-level analysis | yes |
| Specific leaf area | 2012 | high elevation | 2014 | 0.437± 0.223 | 74.5 | 1.96 | 0.054 | 0.087 | yes |  |
| Water-use efficiency (δ13C) | 2012 | low elevation | 2013 | 0.04± 0.398 | 285 | 0.1 | 0.92 | 0.92 | yes |  |
| Water-use efficiency (δ13C) | 2012 | low elevation | 2014 | 0.434± 0.445 | 312 | 0.97 | 0.33 | 0.41 | yes |  |
| Water-use efficiency (δ13C) | 2012 | high elevation | 2013 | 0.181± 0.391 | 268 | 0.46 | 0.64 | 0.69 | yes |  |
| Water-use efficiency (δ13C) | 2012 | high elevation | 2014 | -0.65± 0.466 | 314 | -1.39 | 0.17 | 0.22 | yes |  |
| Height at flowering | 2012 | low elevation | 2013 | -2.07± 0.319 | 289 | -6.47 | <0.0001 | **<0.0001** | yes | yes |
| Height at flowering | 2012 | low elevation | 2014 | -1.78± 0.41 | 590 | -4.35 | 0.00002 | **<0.0001** | yes | yes |
| Height at flowering | 2012 | high elevation | 2013 | -1.75± 0.199 | 48.5 | -8.82 | <0.0001 | **<0.0001** | yes | yes |
| Height at flowering | 2012 | high elevation | 2014 | -0.69± 0.323 | 295 | -2.12 | 0.035 | *0.06* | This cline is not significant in the family-level analysis | yes |
| Flowering phenology (elapsed days since snowmelt) | 2012 | low elevation | 2013 | -1.87± 0.265 | 145 | -7.07 | <0.0001 | **<0.0001** | yes | yes |
| Flowering phenology (elapsed days since snowmelt) | 2012 | low elevation | 2014 | -0.16± 0.332 | 314 | -0.48 | 0.63 | 0.69 | yes |  |
| Flowering phenology (elapsed days since snowmelt) | 2012 | high elevation | 2013 | -1.1± 0.183 | 34.6 | -6.02 | <0.0001 | **<0.0001** | yes | yes |
| Flowering phenology (elapsed days since snowmelt) | 2012 | high elevation | 2014 | -1.87± 0.268 | 150 | -6.99 | <0.0001 | **<0.0001** | yes | yes |

**Table S7: Aster Models and complementary analyses in SAS**

We examine selection via viability, fecundity (among plants that flowered) and cumulative (fecundity among all plants) components of fitness in both Aster and SAS frameworks. To the best of our knowledge, this is the first example of a genotypic selection analysis in Aster that examines fluctuating selection when traits and fitness components differ from year to year. Previous analyses in Aster have estimated phenotypic selection using individual-level data. Nor can we find any published examples of Aster analyses that examine contributions of temporally varying fitness and trait values to cumulative patterns of selection. Rather, previous studies that have multiple years of trait and fitness data appear to link lifetime fitness with trait data averaged across years ([e.g., Colautti and Barrett 2013](#_ENREF_3)). In contrast, we needed to retain temporal differences in trait values across years in our analyses.

In some cases in our dataset, a family may flower in one year, but not another. For those families, we have adult traits (flowering phenology and height at flowering) in only a subset of the years, but we have specific leaf area and δ^13^C along with fitness components in all years. In total, 5 individuals from 5 different families of the 2011 cohort failed to flower in at least one year. For the 2012 cohort, we see a similar issue for one family in the low elevation garden and one family in the high elevation garden. The Aster package discards all records from any family with missing data. Even if a family has a complete set of trait and fitness data in most years of the dataset, the family cannot be used in Aster models if it is missing any trait data from any year. In contrast, repeated measures analysis in SAS does not discard entire families, but only discards the specific years with missing trait data. These families are highly informative in our study because variation in flowering success is targeted by selection, and these families may express trait values that are selected against in a specific location. Because of our novel application of the Aster framework and because SAS is not constrained by missing data, we chose to conduct complementary repeated measures analyses in SAS, which yielded more conservative estimates of selection than Aster did. That is, in some cases, Aster detected patterns of selection that were only marginally significant in SAS. We have labeled those instances as tentative and in need of verification.

“root”

(# plants per genotype

alive in June

2012)

Bernoulli

# plants per genotype

alive in June

2013

# plants per genotype

alive in June

2014

# plants per genotype

that flowered in

2012

# plants per genotype

that flowered in

2013

# plants per genotype

that flowered in

2014

Negative

binomial

average fecundity of individuals that flowered in 2012, rounded to an integer

average fecundity of individuals that flowered in 2013, rounded to an integer

average fecundity of individuals that flowered in 2014, rounded to an integer

Negative

binomial

Negative

binomial

Bernoulli

Bernoulli

Bernoulli

Bernoulli

The graphical model that we used in Aster for the 2011 cohort is to the right. The 2012 cohort followed a similar structure, but modeled fecundity with a normal distribution and included data from the 2013 and 2014 growing seasons only.

**Table S8:** Direct viability, fecundity, and cumulative selection gradients derived from Aster and SAS models for the 2011 cohort. The significance of coefficients for Aster models was assessed with log likelihood ratio tests. We report F-tests from SAS models. Note that standard errors are not straightforward to estimate in Aster analyses. We included source elevationas a covariate in our models to account for unmeasured traits that may also vary across elevational gradients, and we evaluated quadratic effects of source elevation through loglikelihood tests. We standardized all traits to a mean of 0 and standard deviation of 1 and we relativized fecundity to a mean of 1. We converted parameter estimates from logistic regression to selection gradients and differentials following Janzen and Stern ([Janzen and Stern 1998](#_ENREF_4)) by first calcuating the absolute fitness of all families (W), then multiplying logistic regression coefficients and standard errors by the average of W × (1- W), and finally dividing by mean absolute fitness to relativize selection gradients. SAS models evaluated the viability in a logistic regression (Proc Glimmix), and fecundity and cumulative fitness in Poisson regressions (Proc Glimmix) with an R sided random effect (i.e., repeated effect) for genotype with first order autoregression [ar(1)]. Because of the need for Poisson regression, SAS reports hard selection (with absolute fitness as the response variable) for fecundity and cumulative fitness. In contrast, aster reported fecundity and cumulative selection using relative fitness as the response. We estimated standardized linear selection gradients and statistical significance in models that contained linear effects only; we extracted quadratic selection gradients and significance from second order polynomial models including linear and quadratic effects of traits (and other relevant fixed effects) ([Lande and Arnold 1983](#_ENREF_7)). We doubled all quadratic regression coefficients and standard errors to estimate quadratic selection gradients ([Stinchcombe et al. 2008](#_ENREF_8)). Significant selection is highlighted in bold.

|  | 2011 Cohort: Aster | | | | | |
| --- | --- | --- | --- | --- | --- | --- |
|  | Viability | | Fecundity | | Cumulative | |
|  | Linear | Quadratic | Linear | Quadratic | Linear | Quadratic |
| Specific leaf area | 0.283  χ^2^(1)=2.62  p=0.11 | **-1.11**  **χ^2^(1)=32.7**  **p<0.0001** | -0.28  χ^2^(1)=2.44  p=0.12 | **-0.67**  **χ^2^(1)=55.2**  **p<0.0001** | -0.43  χ^2^(1)=0.56  p=0.45 | **-0.77**  **χ^2^(1)=7.5**  **p=0.006** |
| δ^13^C (Water-use efficiency) | **0.180**  **χ^2^(1)=5.31**  **p=0.021** | **-0.36**  **χ^2^(1)=7.2**  **p =0.007** | -0.10  χ^2^(1)=0.09  p=0.76 | **0.001**  **χ^2^(1)=9.4**  **p=0.002** | -0.08  χ^2^(1)=0.03  p=0.87 | -0.06  χ^2^(1)=0.2  p=0.66 |
| Flowering  phenology | 0.130  χ^2^(1)=0.32  p=0.57 | **-0.46**  **χ^2^(1)=8.1**  **p=0.005** | **-0.546**  **χ^2^(1)=64.53**  **p<0.0001** | **0.63**  **χ^2^(1)=17.4**  **p<0.0001** | **-0.74**  **χ^2^(1)=9.08**  **p=0.003** | 0.65  χ^2^(1)=2.46  p=0.12 |
| Height at  flowering | **0.387**  **χ^2^(1)=9.39**  **p=0.002** | **-0.59**  **χ^2^(1)=8.3**  **p=0.004** | **0.41**  **χ^2^(1)=32.6**  **p<0.0001** | -0.24  χ^2^(1)=1.6  p=0.210 | **0.61**  **χ^2^(1)=8.1**  **p=0.005** | -0.10  χ^2^(1)=0.46  p=0.45 |
|  | 2011 Cohort: SAS repeated measures | | | | | |
| Specific leaf area | 0.48 ± 0.25  F_1,35_=3.70  p=0.063 | **-0.81 ± 0.28**  **F_1,31_=8.4**  **p=0.007** | -0.22 ± 0.16  F_1,36_=1.78  p=0.19 | **-1.10 ± 0.26**  **F_1,32_=18.0**  **p<0.0001** | -0.48 ± 0.24  F_1,36_=4.03  p =0.052 | **-1.77 ± 0.43**  **F_1,32_=17.4**  **p=0.0002** |
| δ^13^C (Water-use efficiency) | 0.08 ± 0.137  F_1,35_=0.33  p=0.57 | -0.09 ± 0.18  F_1,31_=0.25  p=0.620 | -0.15 ± 0.09  F_1,36_=2.68  p=0.11 | 0.02 ± 0.14  F_1,32_=0.0  p =0.870 | 0.0004 ± 0.12  F_1,36_=0.0  p =0.997 | -0.19 ± 0.19  F_1,32_=0.9  p=0.34 |
| Flowering  phenology | **-0.45 ± 0.22**  **F_1,35_=4.06**  **p=0.050** | **-0.45 ± 0.19**  **F_1,31_=5.5**  **p=0.025** | **-0.431 ± 0.13**  **F_1,36_=11.08**  **p=0.002** | **0.61 ± 0.14**  **F_1,32_=6.3**  **p =0.018** | **-0.40 ± 0.19**  **F_1,36_=4.61**  **p =0.039** | **0.80 ± 0.32**  **F_1,32_=6.2**  **p=0.018** |
| Height at  flowering | **0.78± 0.17**  **F_1,35=_20.69**  **p<0.0001** | **-0.66 ± 0.22**  **F_1,31_=9.0**  **p=0.005** | **0.29 ± 0.09**  **F_1,36_=9.67**  **p =0.004** | -0.16 ± 0.14  F_1,32_=1.25  p=0.27 | **0.29 ± 0.13**  **F_1,36_=4.65**  **p =0.038** | -0.23 ± 0.21  F_1,32_=1.21  p=0.28 |

**Table S9:** Direct viability, fecundity, and cumulative selection gradients derived from Aster and SAS models for the 2012 cohort in both gardens. The significance of coefficients for Aster models was assessed with log likelihood ratio tests. Formal tests of spatially divergent selection are reported in Table S10. SAS models evaluated the viability in a logistic regression (Proc Glimmix), and fecundity and cumulative fitness using normal distributions (Proc Mixed) with a random effect for genotype with an unstructured covariance matrix. As fecundity and cumulative fitness followed normal distributions, we relativized these terms before analysis and use relative fitness as the response variable. We estimated standardized linear selection gradients and statistical significance in models that contained linear effects only; we extracted quadratic selection gradients and significance from second order polynomial models including linear and quadratic effects of traits (and other relevant fixed effects) ([Lande and Arnold 1983](#_ENREF_7)). We doubled all quadratic regression coefficients and standard errors to estimate quadratic selection gradients ([Stinchcombe et al. 2008](#_ENREF_8)). Significant selection is highlighted in bold.

|  | 2012 Cohort, Low Elevation | | | | | | 2012 Cohort, High Elevation | | | | | |
| --- | --- | --- | --- | --- | --- | --- | --- | --- | --- | --- | --- | --- |
|  | Viability | | Fecundity | | Cumulative | | Viability | | Fecundity | | Cumulative | |
|  | Linear | Quadratic | Linear | Quadratic | Linear | Quadratic | Linear | Quadratic | Linear | Quadratic | Linear | Quadratic |
|  | Aster life history analysis | | | | | | | | | | | |
| Specific leaf area | **-0.18**  χ^2^(1)=0.68  p=0.411 | **0.17**  χ^2^(2)=8.78  p=0.012 | **-0.46**  χ^2^(1)=580.37  p<0.0001 | **0.44**  χ^2^(1)=116.7  p<0.0001 | **-0.31**  χ^2^(2)=26.42  p<0.0001 | 0.46  χ^2^(1)=2.43  p=0.119 | **0.16**  χ^2^(1)=2.72  p=0.100 | **-0.36**  χ^2^(2)=8.78  p=0.012 | **-0.20**  χ^2^(1)=147.8  p<0.0001 | **-0.35**  χ^2^(1)=189.78  p<0.0001 | **-0.33**  χ^2^(2)=26.42  p<0.0001 | **-0.71**  χ^2^(1)=32.35  p<0.0001 |
| δ^13^C (Water-use efficiency) | **0.18**  χ^2^(2)=16.29  p<0.0001 | **0.23**  χ^2^(1)=7.33  p=0.007 | 0.06  χ^2^(1)=3.03  p=0.082 | 0.03  χ^2^(2)=2.71  p=0.258 | 0.01  χ^2^(2)=1.61  p=0.447 | -0.04  χ^2^(1)=0.02  p=0.88 | **0.16**  χ^2^(2)=16.29  p<0.0001 | **-0.30**  χ^2^(1)=3.94  p=0.047 | **-0.12**  χ^2^(1)=76.4  p<0.0001 | 0.02  χ^2^(2)=2.71  p=0.258 | -0.18  χ^2^(2)=1.61  p=0.447 | **-0.23**  χ^2^(1)=16.23  p<0.0001 |
| Flowering  phenology | 0.14  χ^2^(1)=3.39  p=0.07 | **0.46**  χ^2^(1)=7.58  p=0.006 | **-0.49**  χ^2^(1)=675.60  p<0.0001 | **0.26**  χ^2^(1)=45.24  p<0.0001 | **-0.43**  χ^2^(2)=15.09  p<0.0001 | 0.51  χ^2^(1)=0.57  p=0.452 | 0.29  χ^2^(1)=0.29  p=0.588 | **0.19**  χ^2^(1)=7.91  p=0.005 | **-0.18**  χ^2^(1)=79.15  p<0.0001 | **0.09**  χ^2^(1)=36.52  p<0.0001 | **-0.28**  χ^2^(2)=15.09  p<0.0001 | **0.03**  χ^2^(1)=7.31  p=0.007 |
| Height at  flowering | 0.09  χ^2^(1)=0.00  p=0.95 | -0.05  χ^2^(2)=4.93  p=0.085 | **0.10**  χ^2^(1)=45.84  p<0.0001 | **-0.26**  χ^2^(1)=210.2  p<0.0001 | **0.11**  χ^2^(1)=0.92  p=0.339 | -0.24  χ^2^(1)=2.42  p=0.120 | **0.34**  χ^2^(1)=4.80  p=0.029 | 0.09  χ^2^(2)=4.93  p=0.085 | **0.29**  χ^2^(1)=298.9  p<0.0001 | **-0.52**  χ^2^(1)=677.89  p<0.0001 | **0.47**  χ^2^(1)=16.0  p<0.0001 | **-0.81**  χ^2^(1)=46.1  p<0.0001 |
|  | SAS repeated measures analysis | | | | | | | | | | | |
| Specific leaf area | -0.25± 0.17  t_58_=-1.46  p=0.15 | -0.12± 0.43  t_50_=-0.28  p=0.78 | **-0.32**± 0.15  t_58_=-2.17 p=0.034 | 0.50± 0.37  t_50_=-0.27  p=0.18 | **-0.30**± 0.14  t_58_=-2.15 p=0.036 | 0.60±0.35  t_50_=1.69 p=0.097 | 0.12 ± 0.08  t_58_=1.62  p=0.111 | -0.12 ± 0.12  t_50_=-1.02  p=0.31 | -0.23± 0.11  t_58_=-1.97  p=0.053 | -0.35 ± 0.19  t_50_=-1.85 p=0.070 | -0.21 ± 0.11  t_58_=-1.87 p=0.066 | -0.35 ± 0.18  t_50_=-1.95  p=0.057 |
| δ^13^C (Water-use efficiency) | **0.23**± 0.09  t_58_=2.46  p=0.017 | **0.21** ± 0.10  t_49_=2.16 p=0.036 | 0.03± 0.09  t_58_=0.31  p=0.76 | -0.03± 0.09  t_50_=-0.27  p=0.79 | 0.03± 0.08  t_58_=-0.33  p=0.74 | -0.02±0.09  t_50_=-0.17 p=0.86 | 0.11± 0.08  t_58_=1.32  p=0.193 | **-0.24** ± 0.12  t_49_=-2.06  p=0.045 | -0.23 ±0.12  t_58_=-1.82 p=0.074 | -0.05± 0.19  t_50_=-0.24  p=0.815 | -0.19 ± 0.12  t_58_=-1.58 p=0.12 | -0.06 ± 0.19  t_49_=-0.33  p=0.740 |
| Flowering  phenology | **0.44** ± 0.16  t_58_=2.70  p=0.009 | -0.20± 0.43  t_50_=-0.46  p=0.647 | **-0.54**± 0.16  t_58_=-3.35 p=0.001 | 0.50± 0.43  t_50_=1.18  p=0.244 | **-0.51** ±0.15  t_58_=-3.32  p=0.0016 | 0.42±0.41  t_50_=1.02 p=0.31 | **0.57** ± 0.08  t_58_=7.55  p<0.0001 | -0.15± 0.09  t_50_=-1.61  p=0.113 | -0.25 ±0.14  t_58_=-1.82 p=0.075 | 0.09± 0.16  t_50_=0.58  p=0.565 | -0.21± 0.13  t_58_=-1.57 p=0.12 | 0.42 ±0.41  t_50_=1.02  p=0.31 |
| Height at  flowering | -0.05± 0.10  t_58_=-0.45  p=0.653 | 0.19± 0.16  t_50_=1.18  p=0.243 | 0.15± 0.10  t_58_=1.51  p=0.136 | -0.30±0.15  t_50_=-1.97  p=0.055 | 0.12±0.09  t_58_=-1.33  p=0.19 | -0.28±0.15  t_50_=-1.91 p=0.06 | **0.44** ± 0.06  t_58_=7.55  p<0.0001 | -0.08± 0.10  t_50_=-0.82  p=0.419 | **0.33**± 0.10  t_58_=3.18  p=0.002 | **-0.43** ±0.15  t_50_=-2.77  p=0.008 | 0.33 ± 0.10  t_58_=3.33 p=0.0015 | -0.36 ±0.15  t_50_=-2.43  p=0.018 |

**Table S10:** Log-likelihood ratio tests for the significance of linear and quadratic selection gradients derived from Aster models for the 2012 cohort. We include interactions between each trait and garden to test for spatially divergent selection. We report the significance of interactions from full models, and we report the significance of individual traits from models where each trait was retained but its interaction with garden was dropped. In cases where an interaction between a trait and garden was significant, we conducted models of each garden separately to assess the significance of the trait in question. Direct selection gradients are reported in Table S9.

|  | Viability selection | | | | | Fecundity selection | | | | Cumulative selection | | | |
| --- | --- | --- | --- | --- | --- | --- | --- | --- | --- | --- | --- | --- | --- |
|  | Deviance: trait | Deviance: interaction | | Deviance: Low elevation | Deviance: High elevation | Deviance: trait | Deviance: interaction | Deviance: Low elevation | Deviance: High elevation | Deviance: trait | Deviance: interaction | Deviance: Low elevation | Deviance: High elevation |
|  | Linear selection | | | | | | | | | | | | |
| Specific leaf area | **χ^2^(2)=23.5 p<0.0001** | | **χ^2^(1)=15.3**  **p<0.0001** | χ^2^(1)=0.7  p=0.411 | χ^2^(1)=2.7  p=0.10 | **χ^2^(2)=777**  **p<0.0001** | **χ^2^(1)=108**  **p<0.0001** | **χ^2^(1)=580**  **p<0.0001** | **χ^2^(1)=148**  **p<0.0001** | **χ^2^(2)=26.4**  **p<0.0001** | χ^2^(1)=0.60  p=0.439 | -- | -- |
| δ^13^C (Water-use efficiency) | **χ^2^(2)=16.3**  **p<0.0001** | | χ^2^(1)=1.42  p=0.233 | -- | -- | **χ^2^(2)=78.9**  **p<0.0001** | **χ^2^(1)=66.4**  **p<0.0001** | χ^2^(1)=3.03  p=0.082 | **χ^2^(1)=76.4**  **p<0.0001** | χ^2^(2)=1.61  p=0.45 | χ^2^(1)=1.46  p=0.23 | -- | -- |
| Flowering  phenology | **χ^2^(2)=50.4**  **p<0.0001** | | **χ^2^(1)=23.7**  **p<0.0001** | χ^2^(1)=3.4  p=0.07 | χ^2^(1)=0.3  p=0.58 | **χ^2^(2)=795**  **p<0.0001** | **χ^2^(1)=183**  **p<0.0001** | **χ^2^(1)=676**  **p<0.0001** | **χ^2^(1)=79.2**  **p<0.0001** | **χ^2^(2)=15.1**  **p<0.0001** | χ^2^(1)=2.58  p=0.108 | -- | -- |
| Height at  flowering | **χ^2^(2)=86.9**  **p<0.0001** | | **χ^2^(1)=52.6**  **p<0.0001** | χ^2^(1)=0.0  p=0.95 | **χ^2^(1)=4.8**  **p=0.029** | **χ^2^(2)=408**  **p<0.0001** | **χ^2^(1)=88.1**  **p<0.0001** | **χ^2^(1)=45.8**  **p<0.0001** | **χ^2^(1)=299**  **p<0.0001** | **χ^2^(2)=16.9**  **p<0.0001** | **χ^2^(1)=10.8**  **p=0.001** | χ^2^(1)=0.92  p=0.339 | **χ^2^(1)=16.0**  **p<0.001** |
|  | Quadratic selection | | | | | | | | | | | | |
| Specific leaf area | **χ^2^(2)=8.78**  **p=0.012** | | χ^2^(1)=1.58  p=0.208 | -- | -- | **χ^2^(2)=305**  **p<0.0001** | **χ^2^(1)=244**  **p<0.0001** | **χ^2^(1)=117**  **p<0.0001** | **χ^2^(1)=190**  **p<0.0001** | **χ^2^(2)=34.8**  **p<0.0001** | **χ^2^(1)=25.6**  **p<0.0001** | χ^2^(1)=2.43  p=0.119 | **χ^2^(1)=32.4**  **p<0.0001** |
| δ^13^C (Water-use efficiency) | **χ^2^(2)=23.2**  **p<0.0001** | | **χ^2^(1)=22.9**  **p<0.0001** | **χ^2^(1)=7.3**  **p=0.007** | **χ^2^(1)=3.9**  **p=0.047** | χ^2^(2)=2.71  p=0.258 | χ^2^(1)=0.53  p=0.467 | -- | -- | **χ^2^(2)=16.3**  **p<0.0001** | **χ^2^(1)=15.9**  **p<0.0001** | χ^2^(1)=0.02  p=0.88 | **χ^2^(1)=16.2**  **p<0.001** |
| Flowering  phenology | **χ^2^(2)=10.8**  **p=0.005** | | **χ^2^(1)=4.97**  **p=0.026** | **χ^2^(1)=7.6**  **p=0.006** | **χ^2^(1)=7.9**  **p=0.005** | **χ^2^(2)=83.3**  **p<0.0001** | **χ^2^(1)=22.8**  **p<0.0001** | **χ^2^(1)=45.2**  **p<0.0001** | **χ^2^(1)=36.5**  **p<0.0001** | **χ^2^(2)=7.87**  **p=0.020** | **χ^2^(1)=6.00**  **p=0.014** | χ^2^(1)=0.06  p=0.452 | **χ^2^(1)=7.31**  **p=0.007** |
| Height at  flowering | χ^2^(2)=4.93  p=0.085 | | χ^2^(1)=1.45  p=0.228 | -- | -- | **χ^2^(2)=889**  **p<0.0001** | **χ^2^(1)=63.8**  **p<0.0001** | **χ^2^(1)=210**  **p<0.0001** | **χ^2^(1)=678**  **p<0.0001** | **χ^2^(2)=48.5**  **p<0.0001** | **χ^2^(1)=32.1**  **p<0.0001** | χ^2^(1)=2.42  p=0.12 | **χ^2^(1)=46.1**  **p<0.0001** |

**Table S11: Standardized linear and quadratic cumulative selection separately for each year: 2011 cohort.** Here we estimate cumulative selection separately for each growing season of the study. There were three separate years of this study in the low elevation garden (2011 cohort) and two years in the low and high elevation gardens (2012 cohort). We doubled all quadratic estimates and standard errors ([Stinchcombe et al. 2008](#_ENREF_8)). We standardized traits to a mean of 0 and SD of 1 prior to analysis. SLA = Specific leaf area, and WUE = Water-use efficiency, as measured via δ^13^C. Height = height at flowering. Figure 3 depicts linear selection from cumulative selection models, and Fig. S4 depects nonlinear cumulative selection.

**2011 cohort: Cumulative direct selection (Multivariate models)**

|  |  | Linear selection | | | Quadratic selection | | |
| --- | --- | --- | --- | --- | --- | --- | --- |
| Season | Trait | β' ± SE | F-value | p-value | γ' ± SE | F-value | p-value |
| 2012 | SLA | 0.061±0.13 | F_1,15_=0.24 | 0.6325 | -0.766±0.23 | F_1,11_=10.8 | **0.0072** |
| 2012 | WUE | 0.237±0.14 | F_1,15_=2.85 | 0.112 | 0.076±0.2 | F_1,11_=0.15 | 0.7077 |
| 2012 | Flowering phenology | -0.64±0.2 | F_1,15_=10.18 | **0.0061** | -0.732±0.36 | F_1,11_=4.22 | 0.0646 |
| 2012 | Height | 0.56±0.13 | F_1,15_=17.69 | **0.0008** | -0.703±0.31 | F_1,11_=5.03 | **0.0464** |
| 2013 | SLA | 0.021±0.06 | F_1,16_=0.14 | 0.7088 | -1.55±0.19 | F_1,12_=64.93 | **<0.0001** |
| 2013 | WUE | 0.368±0.05 | F_1,16_=54.0 | **<0.0001** | -0.127±0.1 | F_1,12_=1.54 | 0.2386 |
| 2013 | Flowering phenology | -0.292±0.05 | F_1,16_=39.5 | **<0.0001** | 1±0.12 | F_1,12_=66.88 | **<0.0001** |
| 2013 | Height | 0.338±0.05 | F_1,16_=42.2 | **<0.0001** | 0.039±0.18 | F_1,12_=0.05 | 0.8344 |
| 2014 | SLA | -0.658±0.06 | F_1,14_=131.5 | **<0.0001** | -0.713±0.15 | F_1,10_=22.2 | **0.0008** |
| 2014 | WUE | -0.084±0.04 | F_1,14_=4.9 | **0.0439** | -0.438±0.1 | F_1,10_=18.0 | **0.0017** |
| 2014 | Flowering phenology | -0.21±0.04 | F_1,14_=22.0 | **0.0003** | 0.455±0.16 | F_1,10_=8.2 | **0.0168** |
| 2014 | Height | 0.371±0.05 | F_1,14_=50.2 | **<0.0001** | 0.176±0.1 | F_1,10_=3.3 | 0.0974 |

**Table S12: Standardized linear and quadratic cumulative selection separately for each year: 2012 cohort.** Here we estimate cumulative selection separately for both growing seasons of the 2012 cohort. We doubled all quadratic estimates and standard errors ([Stinchcombe et al. 2008](#_ENREF_8)). We standardized traits to a mean of 0 and SD of 1 prior to analysis. SLA = Specific leaf area, and WUE = Water-use efficiency, as measured via δ^13^C. Height = height at flowering. Figure 3 depicts linear selection from cumulative selection models, and Fig. S4 depects nonlinear cumulative selection.

**2012 cohort: Cumulative direct selection (Multivariate models)**

|  |  |  | Linear selection | | | Quadratic selection | | |
| --- | --- | --- | --- | --- | --- | --- | --- | --- |
|  | Garden | Trait | β' ± SE | t_13_ | p-value | γ' ± SE | t_5_ | p-value |
| 2013 | low elevation | Specific leaf area | -0.791±0.32 | -2.61 | **0.0132** ^A^ | -0.479±2.18 | -0.22 | 0.8345 |
|  |  | Water-use efficiency (δ^13^C) | 0.001±0.08 | -0.07 | 0.9463 | -0.147±0.13 | -1.15 | 0.3027 |
|  |  | Flowering phenology | -0.796±0.29 | -2.62 | **0.0128** ^B^ | 0.944±0.86 | 1.1 | 0.3198 |
|  |  | height at flowering | -0.023±0.11 | -0.19 | 0.8486 | -0.261±0.17 | -1.53 | 0.1857 |
|  | high elevation | Specific leaf area | -0.179±0.22 | -1.29 | 0.2063 ^A^ | -0.558±0.4 | -1.4 | 0.2204 |
|  |  | Water-use efficiency (δ^13^C) | 0.092±0.18 | 0.33 | 0.7469 | 0.406±0.4 | 1.01 | 0.3572 |
|  |  | Flowering phenology | -0.303±0.25 | -1.6 | 0.1181 ^B^ | 1.253±0.89 | 1.4 | 0.2196 |
|  |  | height at flowering | 0.032±0.17 | -0.02 | 0.9816 | -0.453±0.27 | -1.69 | 0.1525 |
| 2014 | low elevation | Specific leaf area | -0.373±0.4 | t_12_=-0.93 | 0.3691 | 0.876±0.61 | t_4_=1.44 | 0.2243 |
|  |  | Water-use efficiency (δ^13^C) | 0.54±0.35 | t_12_=1.53 | 0.1523 | -0.475±0.54 | t_4_=-0.88 | 0.4266 |
|  |  | Flowering phenology | -1.52±0.57 | t_12_=-2.68 | 0.0202 | 1.337±1.14 | t_4_=1.17 | 0.3062 |
|  |  | height at flowering | 1.069±0.49 | t_12_=2.16 | 0.0517 ^C^ | 1.129±0.97 | t_4_=1.17 | 0.3073 |
|  | high elevation | Specific leaf area | 0.044±0.46 | t_12_=0.1 | 0.9259 | -0.465±1.03 | t_4_=-0.45 | 0.6739 |
|  |  | Water-use efficiency (δ^13^C) | -0.557±0.39 | t_12_=-1.42 | 0.1803 | -0.464±0.95 | t_4_=-0.49 | 0.6513 |
|  |  | Flowering phenology | 0.004±0.43 | t_12_=0.01 | 0.9924 | -1.059±1.32 | t_4_=-0.8 | 0.4663 |
|  |  | height at flowering | 1.402±0.54 | t_12_=2.59 | 0.0237 ^C^ | -1.754±1.19 | t_4_=-1.47 | 0.2158 |

^A^ There is no interaction between SLA and garden in the overall model. The main effect of SLA was F_1,13_= 5.97, p=0.0296, with a selection gradient of -0.49± 0.20

^B^ There is no interaction between flowering phenology and garden in the overall model. The main effect of flowering phenology was F_1,13_=7.26, p=0.018, with a selection gradient of -0.55± 0.20.

^C^ There is no interaction between height at flowering and garden in the overall model. The main effect of height at flowering was F_1,12_=11.3, p=0.0057, with a selection gradient of 1.24± 0.37

Literature Cited

Anderson, J., Perera, N., Chowdhury, B., and Mitchell-Olds, T. 2015. Microgeographic patterns of genetic divergence and adaptation across environmental gradients in *Boechera stricta* (Brassicaceae). *Am. Nat.* 186:S60-S73.

Benjamini, Y. and Hochberg, Y. 1995. Controlling the false discovery rate: a practical and powerful approach to multiple testing. *Journal of the Royal Statistical Society.* 57:289-300.

Colautti, R. I. and Barrett, S. C. H. 2013. Rapid Adaptation to Climate Facilitates Range Expansion of an Invasive Plant. *Science* 342:364.

Janzen, F. J. and Stern, H. S. 1998. Logistic Regression for Empirical Studies of Multivariate Selection. *Evolution* 52:1564-1571.

Jombart, T. 2008. adegenet: a R package for the multivariate analysis of genetic markers. *Bioinformatics* 24:1403-1405.

Kooyers, N. J., Greenlee, A. B., Coloicchio, J. M., Oh, M., and Blackman, B. K. 2015. Replicate altitudinal clines reveal that evolutionary flexibility underlies adaptation to drought stress in annual *Mimulus guttatus*. *New Phytol.* doi: 10.1111/nph.13153.

Lande, R. and Arnold, S. J. 1983. The measurement of selection on correlated characters. *Evolution* 37.

Stinchcombe, J. R., Agrawal, A. F., Hohenlohe, P. A., Arnold, S. J., and Blows, M. W. 2008. Estimating nonlinear selection gradients using quadratic regression coefficients: Double or nothing? *Evolution* 2435-2440.
